# Seasonal Vitamin D Status in Polish Elite Athletes in Relation to Sun Exposure and Oral Supplementation

**DOI:** 10.1371/journal.pone.0164395

**Published:** 2016-10-12

**Authors:** Jaroslaw Krzywanski, Tomasz Mikulski, Hubert Krysztofiak, Marcel Mlynczak, Ewa Gaczynska, Andrzej Ziemba

**Affiliations:** 1 National Center of Sports Medicine, Warsaw, Poland; 2 Department of Applied Physiology, Mossakowski Medical Research Centre PAS, Warsaw, Poland; 3 Warsaw University of Technology, Faculty of Mechatronics, Institute of Metrology and Biomedical Engineering, Warsaw, Poland; Medical University of Gdańsk, POLAND

## Abstract

Vitamin D does not only influence the musculoskeletal health and mineral homeostasis but it also affects cardiovascular, endocrine, nervous, immune and mental functions, thus it is of considerable importance for both physically active people and elite athletes. However, vitamin D deficiency is common worldwide and results from inadequate endogenous skin synthesis (insufficient ultraviolet B exposure) and diet. To improve the vitamin D status elite athletes often travel to lower latitude during winter. The aim of the study was to evaluate the seasonal vitamin D status in Polish elite athletes according to the sun exposure and oral supplementation. Serum concentration of 25-hydroxyvitamin D (25(OH)D) was measured in the years 2010–2014 in 409 elite athletes, who were divided into the following groups: OUTD—outdoor sports, represented by track and field athletes, who trained in Poland; IND—weightlifters, handball and volleyball players who trained indoors in Poland; SUN—track and field athletes who trained during Polish winter in lower latitude with high sunshine exposure; SUPL—track and field athletes who trained in Poland, had an inadequate vitamin D status (25(OH)D < 30 ng/ml) and were supplemented orally. Inadequate Vitamin D status was observed in 80% of OUTD and 84% of IND athletes in winter, whereas in summer the values amounted to 42% and 83%, respectively. The athletes exposed to sun in winter had significantly higher vitamin D concentration than OUTD group. Oral supplementation improved vitamin D concentration by 45%, whereas winter sun exposure caused its increase by 85%. Except for a few summer months an inadequate status of vitamin D was found in the majority of Polish elite athletes, with the deficiency level being similar to the one observed in non-athletic population. The most serious deficiency was observed in indoor disciplines. Adequate vitamin D status can be achieved by both increased sun exposure, especially in winter, and oral supplementation. Athletes should therefore routinely assess their vitamin D status and be educated how to approach their sunlight exposure, diet and supplementation.

## Introduction

Inadequate vitamin D status is common worldwide and might impact both the athletes’ health and performance [1,2,3,4,5]. Vitamin D is a nutrient that functions as a hormone precursor, which plays a crucial role in the regulation of calcium homeostasis and bone turnover and is therefore essential for musculoskeletal health [6,7,8]. The past two decades has seen a growing body of evidence indicating a possible pleiotropic effect across extraskeletal systems [4,6]. Observational studies have suggested that low vitamin D status is associated with an increased risk of cardiovascular diseases [9], cancers [10], neurocognitive disorders [11], autoimmune diseases [12,13], infections [4,13], mental functions [14] as well as mortality [15]. The dietary source of vitamin D is poor and approx. 90% of its supply originates from sunshine-induced skin synthesis which correlates with latitude, season of the year, time of day, age, skin melanine content and use of sun screens [4,8]. 25-hydroxyvitamin D– 25(OH)D is the best available biomarker of vitamin D status [7,8,16] although there is no consensus about its minimal level required in blood. Low vitamin D status, especially in winter [3,5,17,18,19,20], has been identified in more than a billion people around the world (58–90% of North American and European population) as a consequence of urbanization, indoor lifestyle and sun protection strategies aimed at decreasing the risk of skin cancer incidence. This can be prevented by oral supplementation, food fortification and sensible sun exposure.

The data concerning vitamin D status in athletes are incomplete but the available literature reveals vitamin D deficiency among athletes within the range of 42–83%, which is basically similar to the values observed in sedentary population [2,21,22,23,24]. A recent meta-analysis found that 56% out of 2313 athletes showed inadequate vitamin D status, which positively correlated with higher latitudes, winter and early spring seasons and indoor sports activities [2]. The available data defining the vitamin D status of elite athletes in Poland are also limited [25]. Considering pleiotropic effects of vitamin D the deficient athletes are more subject to the risk of respiratory tract infections, muscle injuries, stress fractures and prolonged recovery [1,21]. Its potential role in athletic performance has not been conclusive yet, there are, however, some data of vitamin D ergogenic potential leading to multiple physiological enhancements [1]. To contribute to our understanding of the issue the assessment of the deficit in athletes according to season, latitude and sport discipline (indoor vs. outdoor) is necessary and should be complemented with consecutive evaluation of the efficacy of possible interventions. This, in turn, could provide the basis for the guidelines for periodization of vitamin D monitoring and strategies (a healthy approach to sunlight exposure, consumption of fortified foods and supplementation with vitamin D preparations) aimed at achieving its proper levels with toxicity prevention included.

## Aim of the Study

The aim of the study was to evaluate the seasonal vitamin D status in Polish elite athletes in relation to the sport discipline and to define the scale of vitamin D deficiency. In athletes with inadequate vitamin D status the effectiveness of recommended oral supplementation was also verified and compared with the exposure to sunlight during summer in Poland and in low-latitude countries during Polish winter.

## Material and Methods

In the present study the following serum 25(OH)D concentration criteria were assumed: <20 ng/ml was defined as deficiency, 21–29 ng/ml as insufficiency, 30–50 ng/ml as normal, 50–100 ng/ml as high and >100 ng/ml as toxic [7,8,26]. Deficiency and insufficiency were classified as inadequate vitamin D status.

The study was carried out in National Center of Sports Medicine during the routine medical monitoring program which consisted of full medical examination and blood analyses including the determination of serum 25(OH)D concentration. The study, including the consent procedure, was approved by the Ethics Committee of Warsaw Medical University (permission AKBE/111/15). As the study was retrospective neither written nor verbal consent for this particular study was obtained, but each subject had signed the consent form for the routine medical monitoring, including the statement of agreement for the use of the results for the scientific purposes. The population of this observational study consisted of 409 international level, male and female Polish athletes (228 males and 181 females). A total of 1271 samples were taken in the morning between 8 and 9 a.m. in different seasons for five consecutive years from 2010 untill 2014. All the athletes were Caucasians with skin type I-III according to Fitzpatrick’s scale [27].

The following groups were distinguished for the purpose of the analysis (Table 1): OUTD—outdoor sports, represented by track and field athletes, who trained in Poland, at the latitude of 49–54° N; IND—weightlifters, handball and volleyball players who trained mostly indoors in Poland; SUN—track and field athletes who trained during Polish winter (Jan-Mar) in The Republic of South Africa (RSA), at the latitude of 27° S and in April on Tenerife, at the latitude of 28° N, and had their samples taken within 1–4 weeks after their return to Poland. Subgroup SUN1 with their samples taken for 25(OH)D to be determined within 1–6 weeks before and 1–4 weeks after the 3–4 weeks’ training camp abroad was distinguished for a separate comparison using the paired statistical analyses. Additionally, SUN2 represented a subgroup of SUN athletes who, after the increased sun exposure during winter camp abroad, had their 25(OH)D concentration determined additionally during consecutive summer in Poland. SUN2 subgroup was used to compare the efficacy of winter exposure to sun during the training camp abroad and summer exposure to sun in Poland. SUPL group was composed of track and field athletes who trained in Poland, had vitamin D deficiency or insufficiency and were supplemented orally. The deficient athletes received individually adjusted vitamin D (cholecalciferol) supplementation of 4000–8000 IU daily, depending on the scale of deficiency, whereas the dose for the insufficient ones was established at 2000 IU in accordance with the experts' guidelines for Poland and Central Europe [26]. The effectiveness of supplementation was evaluated after 8–12 weeks of treatment and only fully compliant athletes who confirmed strict follow-up of the prescribed doses were included in SUPL group. The athletes of the SUN and SUPL groups overlapped OUTD group when they were not training abroad in winter or were not supplemented. 10 a.m.– 1 p.m. and 4–7 p.m. were the usual training hours. During abroad training period (SUN group) the daily sunshine exposure was longer than the one which provides recommended daily skin vitamin D synthesis [7], whereas in OUTD group the exposure duration was weather-dependent.

**Table 1:** Characteristics of subjects.

	n samples	subjects	age [yrs]	BMI [kg∙m^-2^]
OUTD	684	229	25.0 ± 0.2	22.7 ± 0.2
IND	327	178	24.7 ± 0.3	25.2 ± 0.2
SUN	224	102	25.7 ± 0.1	22.2 ± 0.3
SUN1	84	42	25.5 ± 0.6	21.8 ± 0.4
SUN2	48	24	25.7 ± 0.7	21.6 ± 0.6
SUPL	72	36	23.0 ± 0.5	23.2 ± 0.7

Characteristics of all the groups of subjects (mean ± SE). OUTD—track and field athletes who trained in Poland; IND—indoor sports athletes who trained in Poland; SUN—track and field athletes who trained abroad with high sunshine exposure during Polish winter; SUPL—track and field athletes who trained in Poland, had inadequate vitamin D status and were supplemented orally.

Fasting blood samples were collected in the morning from the antecubital vein into the clot activator tubes. The serum concentration of 25(OH)D was determined with Liaison diagnostic system (DiaSorin, Stillwater, MN, USA) by a chemiluminescent immunoassay (CLIA); range of detection 4–150 ng/ml, precision 5.0% CV, accuracy SD 1.2. The laboratory’s precision and accuracy is monthly cross-checked within the group of 264 laboratories—RIQAS. For the purpose of the analysis the seasons were distinguished as whole 3-month periods: winter—beginning of December to the end of February, spring—March to May, summer—June to August, autumn—September to November.

### Statistical analyses

As there were no significant differences depending on sex and BMI, no subgroups according to those criteria were created. The obtained results were analyzed for normality of distribution with Shapiro-Wilk’s test and when the data were not normally distributed Wilcoxon’s test was used to compare groups. The repeated measures analysis by season for the combined groups was performed with ANOVA. Analyses in pairs were conducted in subjects who were measured before and after the training camp abroad, during summer in Poland and after winter sun exposure abroad and before and after oral supplementation. The summer/winter ratio was estimated in subgroups. The values are shown as mean ± SE and *P* value less than 0.05 was considered statistically significant. All statistical analyses were performed using the R software with corresponding packages: ggplot2 and dplyr [28].

## Results

**Fig 1:**
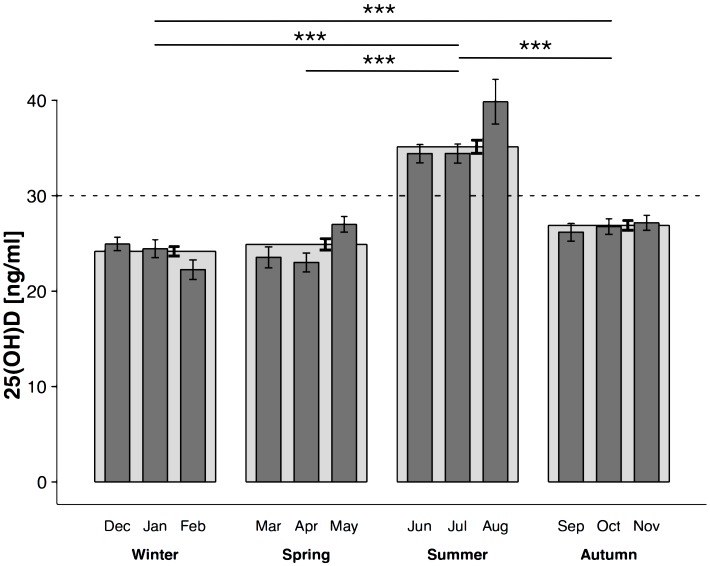
Annual distribution of serum 25(OH)D concentration. Annual distribution of 25(OH)D concentration in serum demonstrated as mean value for consecutive months and seasons in all athletes (mean ± SE, OUTD and IND groups together, n = 1011); the dashed line represents the concentration level considered to be adequate; * denotes a significant difference between summer and other seasons: ***p<0.001.

The annual distribution of 25(OH)D concentration demonstrated as mean value for consecutive months and seasons in all athletes (OUTD and IND groups together) is presented in Fig 1 and all relevant data in S1 Table. From September to May the mean 25(OH)D concentration did not exceed 30 ng/ml. A significant increase and adequate vitamin D status achieved (25(OH)D concentration >30 ng/ml) was found in June (p<0.001) and a decrease was recorded in September (p<0.001). Therefore, only in summer was the adequate vitamin D status achieved and the summer 25(OH)D concentration was significantly higher than in winter, spring and autumn (p<0.001). A significant decrease was observed from autumn to winter (p<0.001) and no change occurred between winter and spring.

**Fig 2:**
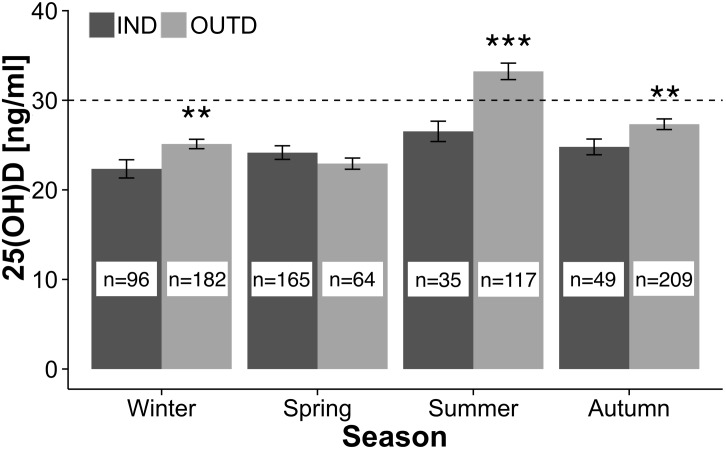
Seasonal variations of serum 25(OH)D concentration in OUTD and IND groups. Seasonal variations of serum 25(OH)D concentration in OUTD and IND groups; the dashed line represents the concentration level considered to be adequate; * denotes significant differences between groups: mean ± SE, **p<0.01, ***p<0.001.

Seasonal concentration of 25(OH)D in OUTD and IND groups and percentages of deficient and insufficient athletes are presented in Table 2 and Fig 2. In OUTD group 25(OH)D concentration was significantly higher in summer than in winter (p<0.001), spring (p<0.001) and autumn (p<0.001) (Table 2). A significant decrease was observed from autumn to winter (p<0.05) and no change occurred between winter and spring. In autumn the 25(OH)D concentration was significantly higher than in spring (p<0.001). In IND group 25(OH)D concentration was significantly higher in summer than in winter (p<0.001) and no changes were identified between summer, spring and autumn. A significantly lower 25(OH)D concentration was found in the athletes of IND group than OUTD group except for spring (in autumn by 10.8%, 3 ng/ml, p<0.01; in winter by 11.2%, 3 ng/ml, p<0.01; and in summer by 26.2%, 9 ng/ml, p<0.001) (Fig 2). Adequate vitamin D status was attained only by the OUTD group athletes in summer.

**Table 2:** Seasonal serum 25(OH)D concentration and percentages of deficient and insufficient athletes.

Group	Winter	Spring	Summer	Autumn
**OUTD (n = 684)**	**25 ± 1 ng/ml**	**26 ± 1 ng/ml**	**36 ± 1 ng/ml**	**28 ± 1 ng/ml**
deficiency	20.7%	17.2%	2.5%	21.4%
insufficiency	59.2%	59.8%	28.6%	45.2%
**IND (n = 327)**	**22 ± 1 ng/ml**	**24 ± 1 ng/ml**	**27 ± 1 ng/ml**	**25 ± 1 ng/ml**
deficiency	42.7%	38.2%	5.7%	22.4%
insufficiency	41.7%	39.4%	51.4%	57.2%

Mean ± SE seasonal 25(OH)D concentration in serum and percentages of deficient (25(OH)D concentration <20 ng/ml) and insufficient (25(OH)D concentration 20–29 ng/ml) athletes in OUTD and IND groups; OUTD—track and field athletes who trained in Poland; IND—indoor sports athletes who trained in Poland.

**Fig 3:**
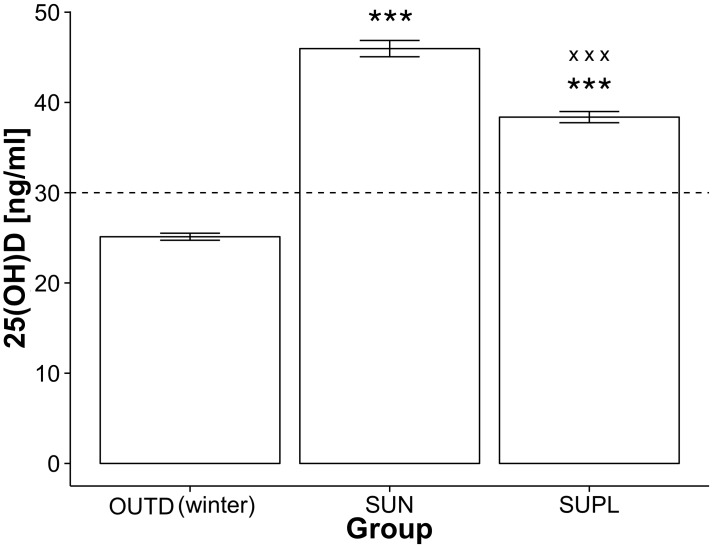
Efficacy of the sun exposition and oral supplementation. The concentration of 25(OH)D in SUN and SUPL groups in comparison with OUTD subjects in winter; mean ± SE, the dashed line represents the concentration level considered to be adequate; * denotes significant differences between OUTD and other groups: *** p<0.001; ^x^ denotes significant differences between SUN and SUPL groups: ^xxx^ p<0.001.

Athletes exposed to sun in winter (SUN group) had a significantly higher 25(OH)D concentration than OUTD group in winter: 46 ± 1 ng/ml vs. 25 ± 1 ng/ml, p<0.001) (Fig 3). Winter exposure to natural sunlight recorded in SUN1 subgroup and expressed as 25(OH)D concentration before and after the overseas training camp resulted in nearly 2-fold increase from 25 ± 1 ng/ml to 46 ± 2 ng/ml, p<0.001. The efficacy of winter sun exposure during the overseas training camp and summer exposure to sun in Poland was compared in SUN2 subgroup and showed a significantly stronger influence of winter sun exposure (44 ± 3 ng/ml vs. 38 ± 2 ng/ml, p<0.01).

The impact of oral supplementation was evaluated in SUPL group and produced a significant increase from 25 ± 1 ng/ml to 37 ± 1 ng/ml, p<0.001.The efficacy analysis of the exposure to sunlight and oral supplementation showed a significant difference between SUN1 and SUPL groups (46 ± 2 ng/ml vs. 37 ± 1 ng/ml, p<0.001). The comparison of differences (deltas) between the efficacy of sun exposure in winter (SUN1) and oral supplementation (SUPL) indicated a significantly stronger influence of sun exposure than oral supplementation (21 ± 2 ng/ml vs. 11 ± 1 ng/ml, p<0.001).

## Discussion

The novel aspect of the present study involves the evaluation of the vitamin D status in as large a group of elite athletes that it can even be comparable to the number of subjects included in meta-analyses. Moreover, the efficacy of oral supplementation versus sun exposure at different latitudes was compared.

### Epidemiology of vitamin D deficiency in athletes

The obtained results revealed the level of vitamin D deficiency among the Polish elite athletes similar to the values described in sedentary population in Poland [19], in other European countries and North America [3,17,18,20] and in athletes [2,21, 22,23,24,25,29]. In the present study 80% of track and field athletes and 84% of indoor athletes had inadequate vitamin D status in winter, whereas in summer the inadequacy pertained to 42% and 83% of the groups respectively. The 25(OH)D concentration was significantly elevated in track and field athletes compared to the athletes practicing indoor sports and such a relationship was observed from summer to winter. Indoor athletes had an inadequate vitamin D status throughout the whole year and even in summer months they did not achieve the required level, with clear seasonal variations maintained. It should be emphasized that in wintertime in both indoor and outdoor athletes the ratio of subjects requiring treatment with vitamin D was similar, whereas in summer it decreased by approx. 50%, though the decrease referred only to track and field athletes. Nevertheless, 25(OH)D concentration monitoring recommended only in wintertime entails the risk that a significant group of athletes, even of outdoor disciplines, will not be provided with proper treatment. Therefore, considering the relatively low cost of testing the athletes, especially the elite group, they should be monitored on a regular basis for the whole year.

Using data from all groups, the difference in 25(OH)D concentration between winter and summer is 8 ng/ml in track and field athletes and 4 ng/ml for indoor athletes with summer to winter ratio 1.32 and 1.19 respectively. In metaanalysis concerning the countries of Central Europe average difference in 25(OH)D between summer and winter was 14–15 ng/ml [5]. In the largest available review of countries above 33° N or S summer to winter difference is 8 ng/ml and the ratio 1.3–1.8 [30,31], what is fully consistent with results of the present study. Comparison of these results demonstrates vitamin D deficit in population of elite athletes similar to the one in overall population. Better vitamin D status is observed in summer in track and field athletes who train mainly outdoor and therefore are more exposed to the sun.

### Seasonal fluctuations

The vitamin D status is influenced by the following factors: latitude, altitude, season, time of the day, air pollution, skin type, area of the skin exposed, sunbathing habits, use of sunscreens, BMI, diet and use of supplements [30,31,32,33]. The significant seasonal differences in vitamin D status observed in our study emphasize the importance of the availability of UVB originating from the sun, which is mostly influenced by the latitude. Poland is situated in moderate climate/latitude between 49° – 54° N, with inadequate sun availability since October to April [30,31,32,33], which constitutes over 6 months' lack of the effective skin synthesis per year. The concentration of 25(OH)D decreases with latitude and in most countries it is 1.5 times higher in summer than in winter. In Boston (USA) at 42° N skin synthesis of vitamin D is ineffective since October to February, and in Edmonton (Canada) at 52°N, Bergen (Norway) at 60° N and Ushuaia (Argentina) at 55° S for full 6 months [32].

In the present study the concentration of 25(OH)D over 30 ng/ml accepted as adequate was found only since June to August with a peak average value recorded in August. Already in September 25(OH)D concentration decreased below that limit and reached its lowest value in spring. These observations are consistent with the results obtained in the USA [34] and in Denmark at the latitude similar to Poland [35]. Consequently, inadequate vitamin D status in track and field athletes was found for 9 months. This could be confusing as the athletes represented the outdoor sport and since March to November most workouts in Poland were conducted outdoors. Possibly, other factors influencing skin synthesis of Vitamin D i.e. sunbathing habits, use of sunscreens or time of the day for training also came into play there [30,31,32,33]. In a study conducted in June in Boston, at the latitude similar to Poland, no vitamin D synthesis was found before 8 a.m. and after 6 p.m., whilst at 8–10 a.m. and 4–6 p.m. it was just 20% of the value observed midday [4]. The highest efficiency of UVB radiation for skin vitamin D synthesis was recorded at 12 p.m. [36]. It should not be forgotten, that between training sessions athletes usually ate and rested indoors, moreover, a remarkable amount of their training sessions, even in the case of runners, took place in a gym.

Bearing in mind that only the morning training session was in the proper sunshine, the conditions provided for effective vitamin D synthesis lasted for only 2–3 hours. Even if the morning training session took place in a gym 2–3 times a week, the total sun exposure met the guidelines criteria (20 min a day between 10 a.m. and 3 p.m. with 20% of skin surface exposed)[7].

Our study subjects practicing indoor disciplines had a permanent inadequate vitamin D status all year round and even in summer their 25(OH)D concentration did not reach the recommended level. Nevertheless, the seasonal rhythm with significant winter-summer fluctuations was still observed. It should be noted that improper vitamin D status in winter was observed in a similar number of athletes of both indoor and outdoor disciplines, whereas in summer vitamin D deficiency was detected in twice as many indoor athletes. In this group a limited access to the UVB radiation caused by training in closed space was an obvious mechanism of impaired vitamin D synthesis.

### Winter sun exposure abroad

In most countries an average summer to winter concentration difference amounts to 8 ng/ml [30,31] and in the present study the value of 8.1 ng/ml was identified in OUTD group, whereas Polish track and field athletes who trained in winter in the lower latitude countries with a higher sun exposure availability (The Republic of South Africa—RSA, Tenerife) showed an increase in average 25(OH)D concentration by 21 ng/ml. The efficacy of vitamin D synthesis after exposure to sun radiation in RSA and Tenerife was higher by 12 ng/ml (37%). Consequently, the sun at the lower latitude, RSA and Tenerife are closer to the equator than Poland by approx. 21°, proved to be a much stronger stimulus for vitamin D synthesis than the sun during summer in Poland. This is consistent with most studies in which the inverse correlation between the latitude and 25(OH)D concentration was described (the average 25(OH)D concentration near the equator amounts to 40 ng/ml, whereas in the far north or south it is just 15 ng/ml) [30,31,32,33]. The ratio between summer UVB radiation at 20° N and 70° N is approx. 2 [30]. The mechanism of more efficient vitamin D synthesis at lower latitudes results from better availability of UVB radiation due to more favorable zenith angle.

### Effectiveness of oral supplementation

In our study oral supplementation of vitamin D in winter increased 25(OH)D concentration to the recommended values. The doses applied according to the guidelines for Central Europe [26] caused the average 25(OH)D concentration to increase by 10 ng/ml. It was previously recorded that the use of 3000 IU daily for 8 weeks produced 25(OH)D concentration of 40–60 ng/ml [32,37]. The efficacy of oral supplementation and the treatment with the doses of 1000–10000 IU daily was also confirmed in several other studies [38,39,40,41]. Oral administration of 100 IU of vitamin D was reported to result in the increase of 25(OH)D concentration by 0.6–1.0 ng/ml [21,40]. When the primary 25(OH)D concentration was lower than 15 ng/ml it was found to increase more rapidly, by 2–3 ng/ml [4,42].

### Oral supplementation vs sun exposure

The comparison of the efficacies of the two applied intervention methods indicates the better results of the increased sun exposure in winter in the latitude/region with higher availability of UVB radiation. It can be explained by a large skin exposure area in track and field athletes who trained in summer conditions during their habitual winter. Higher efficacy of sun exposure than oral supplementation was confirmed in several studies. The exposure of 20% of body surface to sun radiation of 0.5 MED (1 MED—minimum erythema dose—amounts to about 30 minutes of midsummer, midday sun exposure) is an equivalent of an oral dose of 1400–2000 IU of vitamin D [32,33]. The dose of 1 MED applied on the whole body surface produces 10–20000 IU of vitamin D which equals 200–375 ml of cod liver oil [43]. In the present study the highest efficacy of an increase in vitamin D status was found in the group of athletes who were exposed to sun in winter in lower latitudes (RSA and Tenerife). The oral supplementation was also effective and significantly better than the exposure to summer sun in Poland, where only in August was 25(OH)D concentration higher than in the supplemented group. This confirms the weak sun radiation in Poland and Central Europe and ineffective sunshine exposure of athletes. The program for the improvement of vitamin D status is therefore necessary, which particularly pertains to elite athletes.

## Conclusions

To summarize, closer attention should be paid to vitamin D inadequacy in sports medicine. Except for three summer months, not five as described in literature, the majority of Polish elite athletes had inadequate status of vitamin D, which may negatively impact their health and performance. Furthermore, the prevention strategies and personalized treatment of elite athletes are of higher significance than in non-athletic population. Their vitamin D status should therefore be monitored regularly. Athletes of indoor disciplines are particularly prone to vitamin D deficiency all year round and they require particularly careful attention. An adequate vitamin D status can be achieved by both increased sun exposure and oral supplementation. However, winter sun exposure abroad in lower latitude locations proves to be more efficient than oral supplementation with recommended doses applied in the present study as well as more efficient than summertime sun exposure in Poland.

## Supporting Information

S1 Table The serum 25(OH)D concentration of all examined athletes. Consecutive values of all the performed measurements throughout the whole study period.
(PDF)
