# Supplementary material for: Seasonal Vitamin D Status in Polish Elite Athletes in Relation to Sun Exposure and Oral Supplementation
Source: PLoS One. 2016 Oct 12;11(10):e0164395. doi: 10.1371/journal.pone.0164395 (PMC5061377; doi:10.1371/journal.pone.0164395)
Supplement: S1 Table — Consecutive values of all the performed measurements throughout the whole study period. (PDF) [file pone.0164395.s001.pdf]

No. of person	Sex	Month of measure	Year of measure	Age	BMI	25(OH)D Level	Supplementation	Preceding camp	Discipline type
1	male	10	2012	17	24,19	24,10	0	0	Out
1	male	1	2013	18	24,19	27,80	2	0	Out
2	male	6	2012	25	23,31	34,50	0	1	Out
2	male	7	2012	25	23,31	35,30	0	1	Out
2	male	1	2013	26	23,31	25,30	1	0	Out
2	male	7	2014	27	23,31	42,70	0	0	Out
3	female	10	2014	24	19,51	25,10	0	0	Out
4	male	11	2010	26	23,10	18,90	0	0	Out
4	male	2	2011	27	23,10	32,80	2	0	Out
4	male	5	2011	27	23,04	30,40	2	0	Out
4	male	1	2013	29	23,04	26,50	1	0	Out
4	male	6	2013	29	23,04	28,00	0	0	Out
5	female	11	2012	20	22,00	28,90	0	0	Out
5	female	1	2013	21	22,00	37,60	2	0	Out
5	female	2	2013	21	22,00	37,00	2	0	Out
5	female	3	2013	21	22,00	40,00	2	0	Out
5	female	12	2013	21	22,00	23,00	0	0	Out
5	female	1	2014	22	22,00	43,00	2	0	Out
5	female	6	2014	22	22,00	28,50	0	0	Out
5	female	12	2014	22	22,00	34,30	2	0	Out
6	female	6	2012	23	18,31	31,00	0	1	Out
6	female	7	2012	23	18,31	32,00	0	1	Out
6	female	2	2013	24	18,31	33,00	1	0	Out
6	female	10	2014	25	18,31	20,00	0	0	Out
7	female	12	2009	20	19,38	28,57	0	0	Out
7	female	5	2010	22	19,38	54,10	1	1	Out
7	female	7	2010	22	19,05	26,09	0	1	Out
7	female	12	2010	22	19,18	22,46	0	0	Out
7	female	12	2011	23	18,78	28,90	0	0	Out
7	female	6	2012	24	18,78	37,70	0	1	Out
7	female	11	2012	24	18,78	37,10	1	0	Out
7	female	2	2013	25	18,78	55,00	1	0	Out
7	female	6	2013	25	18,78	43,00	1	1	Out
7	female	7	2013	25	18,78	50,00	0	1	Out
7	female	12	2013	25	18,78	29,00	0	0	Out
7	female	6	2014	26	18,78	36,80	0	0	Out
7	female	7	2014	26	18,78	36,70	0	0	Out
7	female	12	2014	26	18,78	31,90	2	0	Out
7	female	1	2015	27	18,78	55,20	1	0	Out
8	male	5	2010	23	22,09	36,59	1	0	Out

8	male	7	2010	23	22,09	27,15	0	1	Out
8	male	2	2011	24	21,97	21,10	0	0	Out
8	male	11	2011	24	21,92	28,90	0	0	Out
9	female	4	2012	23	17,67	22,70	0	0	Out
10	female	2	2012	20	19,59	13,90	0	0	Out
10	female	9	2013	21	19,59	26,00	0	0	Out
10	female	10	2013	21	19,59	38,00	2	0	Out
10	female	1	2014	22	19,59	42,00	2	0	Out
10	female	6	2014	22	19,59	28,80	0	0	Out
10	female	10	2014	22	19,59	33,80	0	0	Out
11	male	2	2011	20	25,00	34,10	0	0	Out
11	male	5	2011	20	25,00	54,10	1	0	Out
12	male	5	2011	27	22,77	28,70	1	0	Out
12	male	1	2015	31	22,77	29,30	0	0	Out
13	male	8	2011	18	23,95	27,30	0	0	Out
14	male	5	2014	24	21,05	25,70	0	0	Out
15	male	1	2013	21	24,04	24,40	0	0	Out
16	female	6	2011	21	18,87	69,90	1	1	Out
16	female	12	2011	21	18,83	40,80	0	0	Out
16	female	4	2013	23	18,83	40,00	1	1	Out
16	female	12	2013	23	18,83	29,00	0	0	Out
16	female	2	2015	25	18,83	26,50	0	0	Out
17	female	11	2009	23	19,03	25,76	0	0	Out
17	female	4	2010	24	19,72	59,38	1	0	Out
17	female	6	2010	24	19,38	30,08	0	1	Out
17	female	7	2010	24	19,38	25,66	0	1	Out
17	female	11	2010	24	19,72	20,40	0	0	Out
17	female	3	2011	25	19,72	23,70	0	0	Out
18	male	11	2010	25	25,67	15,69	0	0	Out
18	male	12	2010	25	25,67	21,10	0	0	Out
18	male	2	2011	26	25,35	33,70	1	0	Out
18	male	5	2011	26	24,51	30,20	0	1	Out
18	male	11	2011	26	24,38	31,90	0	0	Out
18	male	3	2013	28	24,38	39,00	2	0	Out
19	male	4	2013	20	36,33	16,00	0	0	Out
20	male	6	2012	24	23,97	38,30	1	1	Out
21	female	11	2012	26	17,23	20,80	0	0	Out
21	female	4	2013	27	17,23	32,00	1	0	Out
21	female	12	2013	27	17,23	19,00	0	0	Out
22	male	11	2010	24	18,77	17,93	0	0	Out
22	male	6	2012	26	18,77	26,70	0	0	Out

22	male	5	2014	28	18,77	29,20	0	0	Out
23	female	10	2013	17	19,14	38,00	0	0	Out
24	male	7	2014	25	27,95	31,30	0	0	Out
25	female	11	2009	32	19,32	54,81	2	0	Out
25	female	10	2010	33	18,71	18,17	0	0	Out
25	female	4	2011	34	36,10	39,70	1	1	Out
25	female	12	2011	34	18,98	21,80	0	0	Out
25	female	3	2012	35	18,98	29,40	1	0	Out
25	female	4	2012	35	18,98	29,40	1	1	Out
25	female	6	2012	35	18,98	41,30	1	1	Out
26	female	10	2010	24	21,70	19,47	0	0	Out
26	female	1	2011	25	21,63	34,80	1	0	Out
26	female	2	2011	25	21,63	38,60	1	1	Out
27	female	6	2010	22	19,60	32,50	0	1	Out
27	female	4	2011	23	19,07	38,10	1	1	Out
27	female	6	2011	23	19,07	37,80	0	1	Out
27	female	11	2011	23	20,07	41,30	0	0	Out
27	female	6	2012	24	20,07	51,20	0	1	Out
27	female	11	2012	24	20,07	38,30	0	0	Out
27	female	4	2013	25	20,07	36,00	1	1	Out
27	female	10	2013	25	20,07	36,00	0	0	Out
27	female	12	2013	25	20,07	53,00	1	0	Out
27	female	11	2014	26	20,07	30,80	0	0	Out
27	female	12	2014	26	20,07	39,50	2	0	Out
28	male	12	2009	21	23,10	25,00	0	0	Out
28	male	4	2010	22	23,10	22,52	0	0	Out
28	male	10	2010	22	23,10	17,00	0	0	Out
28	male	2	2011	23	24,00	32,80	1	0	Out
28	male	3	2011	23	23,88	28,30	0	1	Out
28	male	7	2011	22	23,04	31,50	0	0	Out
28	male	10	2011	22	23,04	23,90	0	0	Out
28	male	11	2011	22	23,04	28,00	0	0	Out
28	male	12	2011	22	23,04	23,80	0	0	Out
28	male	6	2012	23	23,04	19,80	0	0	Out
28	male	1	2013	24	23,04	33,70	2	0	Out
28	male	2	2013	24	23,04	46,00	2	0	Out
29	male	7	2012	24	34,30	40,00	0	1	Out
29	male	1	2013	25	34,30	27,30	0	0	Out
29	male	2	2014	26	34,30	43,00	1	0	Out
30	male	2	2011	22	21,60	66,70	1	0	Out
30	male	4	2011	22	21,60	45,80	1	1	Out

31	male	12	2010	27	25,36	19,40	0	0	Out
31	male	6	2012	29	25,36	35,20	0	1	Out
31	male	2	2013	30	25,36	40,00	1	0	Out
31	male	11	2014	31	25,36	29,70	0	0	Out
32	male	2	2013	21	21,11	14,00	0	0	Out
32	male	4	2013	21	21,11	32,00	2	0	Out
33	male	10	2010	27	21,86	20,84	0	0	Out
33	male	3	2011	28	22,25	20,40	0	0	Out
34	male	11	2010	24	22,14	17,78	0	0	Out
34	male	2	2011	25	21,47	40,30	1	0	Out
34	male	3	2011	25	21,47	29,80	0	1	Out
34	male	2	2015	29	21,47	27,90	0	0	Out
35	male	2	2011	24	22,88	32,40	1	0	Out
35	male	8	2011	24	22,16	32,40	0	0	Out
35	male	10	2011	24	22,16	35,90	0	0	Out
35	male	2	2012	25	22,16	26,20	0	0	Out
36	male	10	2013	19	21,22	27,00	0	0	Out
36	male	6	2014	20	21,22	36,50	0	0	Out
36	male	2	2015	21	21,22	38,60	1	0	Out
37	female	2	2010	28	19,49	24,93	0	0	Out
37	female	11	2010	29	19,49	20,06	0	0	Out
37	female	4	2011	30	20,48	31,20	0	1	Out
37	female	11	2011	30	20,15	37,50	0	0	Out
37	female	5	2012	31	20,15	49,60	0	1	Out
37	female	2	2013	32	20,15	29,00	0	1	Out
37	female	12	2013	32	20,15	30,00	0	0	Out
37	female	2	2014	33	20,15	26,00	0	0	Out
37	female	6	2014	33	20,15	31,10	0	0	Out
38	male	3	2012	25	24,46	21,20	0	0	Out
39	male	4	2010	34	19,46	30,37	0	0	Out
40	male	7	2010	27	23,27	30,80	0	0	Out
40	male	1	2011	28	23,24	29,40	0	0	Out
40	male	3	2011	28	23,24	38,50	1	0	Out
41	female	3	2012	27	19,74	41,50	1	1	Out
41	female	4	2012	27	19,74	30,50	1	1	Out
41	female	6	2012	27	19,74	32,20	2	0	Out
41	female	11	2012	27	19,74	20,70	0	0	Out
41	female	1	2013	28	19,74	35,80	2	0	Out
41	female	3	2013	28	19,74	29,00	2	0	Out
41	female	4	2013	28	19,74	33,00	2	0	Out
41	female	7	2013	28	19,74	46,00	2	0	Out

41	female	11	2013	28	19,74	24,00	0	0	Out
41	female	4	2014	29	19,74	47,00	1	0	Out
41	female	6	2014	29	19,74	41,00	2	0	Out
41	female	12	2014	29	19,74	34,10	2	0	Out
42	female	11	2010	16	17,47	42,22	0	0	Out
43	female	12	2009	23	19,38	33,60	1	0	Out
43	female	2	2011	25	19,38	42,10	1	0	Out
43	female	3	2011	25	19,38	30,60	0	1	Out
43	female	4	2012	26	19,38	56,50	1	1	Out
43	female	10	2012	26	19,38	40,30	0	0	Out
43	female	4	2013	27	19,38	24,00	0	0	Out
44	female	2	2015	20	17,10	39,10	0	0	Out
45	male	1	2010	21	32,90	27,77	0	0	Out
45	male	11	2010	21	34,25	18,23	0	0	Out
45	male	6	2011	22	34,22	32,60	0	1	Out
45	male	1	2012	23	34,22	29,80	0	0	Out
45	male	2	2012	23	34,22	64,70	1	1	Out
45	male	11	2012	23	34,22	30,10	0	0	Out
45	male	9	2013	24	34,22	27,00	0	0	Out
45	male	10	2013	24	34,22	34,00	2	0	Out
45	male	11	2014	25	34,22	27,80	0	0	Out
46	male	1	2010	30	20,90	21,74	0	0	Out
46	male	4	2011	31	20,20	39,30	1	1	Out
46	male	11	2011	31	21,36	30,00	0	0	Out
46	male	12	2012	32	21,36	25,70	0	0	Out
46	male	1	2013	33	21,36	20,80	0	0	Out
46	male	11	2014	34	21,36	29,30	0	0	Out
47	female	6	2012	23	34,64	35,00	0	1	Out
47	female	10	2012	23	34,64	31,10	0	0	Out
47	female	1	2013	24	34,64	34,40	2	0	Out
47	female	9	2013	24	34,64	19,00	0	0	Out
47	female	4	2014	25	34,64	29,00	1	0	Out
47	female	10	2014	25	34,64	21,70	0	0	Out
48	male	2	2013	22	20,39	14,00	0	0	Out
49	female	12	2009	32	19,39	26,67	0	0	Out
49	female	7	2010	33	19,47	27,44	0	0	Out
49	female	6	2011	34	19,00	63,60	0	1	Out
49	female	7	2011	34	19,00	49,10	0	1	Out
50	male	11	2010	25	41,04	19,05	0	0	Out
50	male	5	2011	26	40,61	24,50	0	0	Out
50	male	6	2012	27	40,61	36,00	0	1	Out

50	male	12	2012	27	40,61	31,00	2	0	Out
51	male	12	2009	28	19,68	26,51	0	0	Out
51	male	2	2011	30	17,26	16,90	0	0	Out
51	male	8	2011	31	20,43	35,40	0	0	Out
51	male	12	2012	32	20,43	20,70	0	0	Out
52	female	12	2009	26	28,20	26,51	0	0	Out
52	female	6	2010	27	27,31	24,33	0	0	Out
52	female	11	2010	27	27,32	26,83	0	0	Out
52	female	6	2011	28	26,36	39,70	0	1	Out
52	female	11	2011	28	27,76	50,30	2	0	Out
52	female	6	2012	29	27,76	44,40	0	1	Out
52	female	2	2013	30	27,76	56,00	1	0	Out
52	female	12	2013	30	27,76	27,00	0	0	Out
52	female	12	2014	31	27,76	34,30	2	0	Out
53	female	10	2010	18	19,49	18,49	0	0	Out
53	female	6	2012	20	19,49	26,00	0	1	Out
53	female	7	2012	20	19,49	28,60	0	0	Out
53	female	2	2013	21	19,49	13,00	0	0	Out
53	female	4	2013	21	19,49	39,00	1	0	Out
53	female	12	2013	21	19,49	15,00	0	0	Out
53	female	8	2014	22	19,49	32,50	0	0	Out
54	male	5	2014	24	25,83	54,10	2	0	Out
54	male	12	2014	24	25,83	36,60	0	0	Out
55	male	2	2011	21	25,83	12,23	0	0	Out
55	male	4	2012	22	25,83	23,90	0	0	Out
55	male	12	2012	22	25,83	29,70	2	0	Out
55	male	1	2013	23	25,83	30,30	2	0	Out
55	male	5	2013	23	25,83	46,00	2	0	Out
56	female	11	2010	19	33,12	16,23	0	0	Out
56	female	5	2011	20	32,45	20,70	0	0	Out
56	female	11	2011	20	33,09	25,70	0	0	Out
56	female	5	2012	21	33,09	21,60	0	0	Out
56	female	12	2012	21	33,09	17,90	0	0	Out
56	female	6	2013	22	33,09	23,00	0	0	Out
56	female	6	2014	23	33,09	18,10	0	0	Out
57	female	2	2012	32	20,55	7,90	0	0	Out
58	male	12	2012	21	22,13	19,10	0	0	Out
58	male	4	2013	22	22,13	38,00	2	0	Out
59	male	6	2011	21	22,86	43,70	0	0	Out
60	male	2	2015	23	34,23	11,80	0	0	Out
61	female	2	2013	17	23,60	21,00	0	0	Out

61	female	3	2014	18	23,60	18,00	0	0	Out
62	male	2	2012	23	22,18	36,50	1	0	Out
62	male	12	2012	23	22,18	31,00	0	0	Out
62	male	12	2014	25	22,18	23,00	0	0	Out
63	female	1	2013	21	19,49	36,00	1	0	Out
63	female	7	2013	21	19,49	29,00	0	1	Out
63	female	12	2013	21	19,49	23,00	0	0	Out
63	female	5	2014	22	19,49	42,30	2	0	Out
63	female	8	2014	22	19,49	38,90	0	0	Out
63	female	12	2014	22	19,49	20,00	0	0	Out
63	female	1	2015	23	19,49	34,90	1	0	Out
64	female	11	2010	20	19,63	22,60	0	0	Out
64	female	6	2012	22	19,63	51,60	0	1	Out
65	male	11	2010	27	26,65	30,27	0	0	Out
65	male	7	2011	28	25,63	62,70	0	1	Out
65	male	1	2012	29	25,63	45,70	1	0	Out
65	male	11	2012	29	25,63	54,40	0	0	Out
65	male	1	2013	30	25,63	40,50	1	0	Out
66	male	4	2010	20	21,74	31,03	0	0	Out
66	male	10	2010	20	20,53	18,21	0	0	Out
66	male	1	2011	21	20,53	37,50	2	0	Out
66	male	3	2011	21	21,13	33,30	0	0	Out
66	male	4	2011	21	21,13	48,70	2	0	Out
66	male	6	2011	21	21,13	42,50	2	0	Out
66	male	10	2014	24	21,13	35,90	0	0	Out
66	male	1	2015	25	21,13	47,18	1	0	Out
67	female	12	2009	28	22,72	31,48	0	0	Out
67	female	9	2010	29	22,24	27,16	0	0	Out
67	female	11	2011	29	24,42	33,80	0	0	Out
68	female	6	2011	20	19,82	41,30	0	0	Out
69	female	12	2014	19	19,37	20,30	0	0	Out
70	male	6	2012	20	22,03	68,40	0	1	Out
70	female	6	2013	21	22,03	53,00	0	1	Out
71	male	7	2010	25	20,16	24,52	0	0	Out
71	male	10	2010	25	20,16	24,05	0	0	Out
71	male	4	2011	26	19,72	67,90	1	0	Out
71	male	1	2013	28	19,72	33,10	1	0	Out
71	male	7	2013	28	19,72	31,00	0	0	Out
71	male	12	2013	28	19,72	28,00	0	0	Out
72	female	1	2010	24	20,94	36,21	0	0	Out
72	female	5	2010	10	20,94	54,40	1	1	Out

72	female	6	2010	24	20,94	35,45	0	1	Out
72	female	7	2010	24	20,94	24,26	0	1	Out
72	female	1	2011	25	21,12	20,43	0	0	Out
72	female	5	2011	26	21,30	54,70	1	1	Out
72	female	6	2011	26	21,30	45,30	0	1	Out
72	female	7	2011	25	21,30	31,10	0	1	Out
72	female	11	2011	24	21,12	19,15	0	0	Out
72	female	12	2011	25	21,49	37,00	0	0	Out
72	female	4	2012	26	20,94	58,10	1	1	Out
72	female	7	2012	26	21,49	45,10	0	1	Out
72	female	1	2013	27	21,49	30,80	1	0	Out
72	female	4	2013	27	21,49	43,00	1	1	Out
72	female	11	2014	28	20,94	29,30	0	0	Out
73	female	5	2009	31	19,66	35,83	1	1	Out
73	female	12	2009	31	20,55	24,04	0	0	Out
73	female	6	2010	32	19,84	35,83	0	1	Out
73	female	7	2010	32	19,84	28,26	0	1	Out
73	female	9	2010	32	20,02	22,67	0	0	Out
73	female	10	2010	32	20,02	18,91	0	0	Out
73	female	12	2010	32	20,02	16,61	0	0	Out
73	female	3	2011	33	20,02	82,00	1	1	Out
73	female	4	2011	33	20,02	72,80	1	1	Out
73	female	8	2011	33	20,02	40,20	0	0	Out
73	female	10	2011	33	20,02	34,20	0	0	Out
73	female	1	2012	34	20,02	31,50	1	0	Out
73	female	3	2012	34	20,02	52,40	1	1	Out
73	female	6	2012	34	20,02	42,30	0	1	Out
73	female	7	2012	34	20,02	37,40	0	1	Out
73	female	12	2012	34	20,02	24,40	0	0	Out
73	female	5	2013	35	20,02	20,00	0	0	Out
74	female	1	2010	28	20,67	34,68	0	0	Out
74	female	5	2010	28	20,67	63,95	1	1	Out
75	male	12	2012	19	19,76	20,40	0	0	Out
76	female	1	2012	21	15,98	44,90	2	0	Out
76	female	11	2012	21	15,98	39,50	2	0	Out
76	female	5	2013	22	15,98	33,00	2	0	Out
76	female	10	2013	22	15,98	33,00	0	0	Out
76	female	11	2013	22	15,98	32,00	0	0	Out
76	female	6	2014	23	15,98	26,00	0	0	Out
76	female	10	2014	23	15,98	43,60	0	0	Out
76	female	11	2014	23	15,98	31,40	0	0	Out

76	female	12	2014	23	15,98	29,40	2	0	Out
76	female	2	2015	24	15,98	42,00	1	0	Out
77	female	9	2014	25	23,62	28,90	0	0	Out
78	female	1	2013	20	20,67	54,50	0	0	Out
79	male	12	2014	21	29,33	13,90	0	0	Out
80	female	6	2011	21	19,43	31,00	0	0	Out
81	female	9	2013	26	18,33	32,00	0	0	Out
81	female	3	2014	27	18,33	31,00	2	0	Out
81	female	10	2014	27	18,33	23,00	0	0	Out
82	female	11	2010	18	23,87	10,46	0	0	Out
83	male	11	2009	25	22,44	28,66	0	0	Out
83	male	4	2010	26	23,08	32,78	1	0	Out
84	male	6	2011	22	20,30	25,50	0	0	Out
84	male	4	2012	23	20,30	22,30	0	0	Out
84	male	1	2013	24	20,30	20,90	0	0	Out
84	male	2	2013	24	20,30	25,00	0	0	Out
84	male	7	2013	24	20,30	23,00	0	0	Out
84	male	7	2014	25	20,30	27,10	0	0	Out
84	male	2	2015	26	20,30	14,40	0	0	Out
85	female	1	2010	20	18,51	27,15	0	0	Out
85	female	2	2010	20	18,51	17,57	0	0	Out
85	female	4	2010	20	18,51	32,28	1	1	Out
85	female	6	2010	20	18,69	25,63	0	1	Out
85	female	7	2010	20	18,69	25,74	0	0	Out
85	female	8	2010	20	18,69	27,30	0	0	Out
85	female	10	2010	20	18,44	22,16	0	0	Out
85	female	1	2011	21	19,38	22,87	0	0	Out
85	female	2	2011	21	19,38	62,40	1	0	Out
85	female	3	2011	21	19,38	36,32	0	1	Out
85	female	4	2011	21	19,38	54,10	1	1	Out
85	female	5	2011	21	19,38	28,40	0	1	Out
85	female	6	2011	21	19,38	40,90	0	0	Out
85	female	7	2011	21	19,38	34,40	0	0	Out
85	female	9	2011	21	19,38	37,00	0	0	Out
85	female	11	2011	21	19,38	38,10	2	0	Out
85	female	1	2012	22	19,38	32,90	2	0	Out
85	female	1	2012	22	19,38	39,20	2	0	Out
85	female	4	2012	22	19,38	53,80	2	1	Out
85	female	5	2012	22	19,38	50,70	0	1	Out
85	female	6	2012	22	19,38	40,90	0	1	Out
85	female	7	2012	22	19,38	39,50	0	0	Out

85	female	10	2012	22	19,38	24,10	0	0	Out
85	female	1	2013	23	19,38	26,60	0	0	Out
85	female	4	2013	23	19,38	30,00	2	0	Out
85	female	11	2013	23	19,38	36,00	2	0	Out
85	female	4	2014	24	19,38	38,90	2	0	Out
85	female	6	2014	24	19,38	40,00	0	0	Out
85	female	10	2014	24	19,38	30,00	0	0	Out
86	male	11	2009	29	25,68	25,09	0	0	Out
86	male	4	2010	30	26,22	32,69	1	0	Out
86	male	11	2010	30	26,13	33,82	0	0	Out
86	male	2	2011	31	25,68	31,40	0	0	Out
86	male	3	2011	31	25,68	22,80	0	0	Out
87	male	11	2010	26	20,78	15,90	0	0	Out
87	male	2	2011	27	20,78	15,90	0	0	Out
88	female	10	2010	18	20,24	14,11	0	0	Out
88	female	7	2011	19	19,95	28,90	0	0	Out
88	female	10	2011	19	19,95	22,90	0	0	Out
89	female	6	2011	18	17,46	38,80	0	1	Out
89	female	3	2012	19	17,46	28,20	0	0	Out
89	female	4	2013	20	17,46	24,00	0	0	Out
89	female	11	2013	20	17,46	31,00	0	0	Out
89	female	5	2014	21	17,46	33,90	0	0	Out
89	female	12	2014	21	17,46	29,30	0	0	Out
89	female	1	2015	22	17,46	34,90	2	0	Out
90	female	6	2012	17	19,34	22,10	0	0	Out
91	male	12	2011	21	28,82	25,10	0	0	Out
92	female	1	2010	29	21,18	30,44	0	0	Out
92	female	7	2010	29	22,99	36,47	0	0	Out
92	female	1	2011	30	22,99	22,90	0	0	Out
92	female	5	2011	30	21,44	23,90	0	0	Out
92	female	11	2011	30	21,18	36,20	2	0	Out
92	female	7	2012	30	21,18	35,40	0	0	Out
93	female	2	2013	27	18,98	21,00	0	0	Out
94	female	12	2009	20	20,52	36,57	0	0	Out
94	female	5	2010	21	20,39	43,63	1	0	Out
94	female	7	2010	21	20,39	36,47	0	0	Out
94	female	12	2010	21	20,39	18,79	0	0	Out
94	female	1	2011	22	20,39	31,80	1	0	Out
94	female	6	2011	22	20,39	67,60	0	1	Out
94	female	1	2013	24	20,39	53,30	1	0	Out
95	female	12	2009	24	17,17	30,11	0	0	Out

95	female	4	2010	25	17,98	36,57	1	1	Out
95	female	10	2010	25	16,92	24,18	0	0	Out
95	female	1	2011	26	16,92	35,80	1	0	Out
95	female	3	2011	26	16,92	47,10	1	1	Out
95	female	6	2011	26	17,40	41,10	0	1	Out
95	female	3	2012	27	17,40	57,10	1	1	Out
95	female	6	2012	27	17,40	53,60	0	1	Out
95	female	1	2013	28	17,40	28,10	1	0	Out
95	female	5	2013	28	17,40	21,00	0	0	Out
95	female	6	2014	29	17,40	23,60	0	0	Out
95	female	12	2014	29	17,40	24,40	0	0	Out
96	male	10	2013	28	42,67	17,00	0	0	Out
96	male	12	2013	28	42,67	32,00	2	0	Out
97	male	11	2009	23	21,78	31,88	0	0	Out
97	male	4	2010	24	22,09	52,33	1	1	Out
97	male	10	2010	24	22,09	29,70	0	0	Out
97	male	2	2011	25	22,09	24,30	0	0	Out
97	male	4	2011	25	22,09	85,10	1	0	Out
97	male	7	2011	25	22,09	45,50	0	1	Out
97	male	11	2011	25	22,35	47,70	0	0	Out
97	male	4	2012	26	22,35	56,30	1	1	Out
97	male	11	2012	25	22,35	36,20	0	0	Out
97	male	4	2013	25	22,35	57,00	1	1	Out
97	male	11	2013	25	22,35	28,00	0	0	Out
97	male	12	2014	26	23,29	29,60	2	0	Out
98	male	2	2011	22	22,27	34,00	0	0	Out
98	male	3	2011	23	22,27	22,60	0	0	Out
98	male	5	2012	24	22,27	53,60	1	1	Out
98	male	1	2013	25	22,27	43,90	1	0	Out
98	male	3	2013	25	22,27	41,00	1	1	Out
98	male	5	2013	25	22,27	38,00	1	1	Out
98	male	12	2013	25	22,27	31,00	0	0	Out
98	male	4	2014	26	22,27	30,70	0	0	Out
98	male	6	2014	26	22,27	33,00	0	0	Out
98	male	10	2014	26	22,27	38,90	0	0	Out
98	male	1	2015	25	22,27	64,00	1	0	Out
99	male	4	2012	20	27,98	29,50	0	0	Out
99	male	12	2012	20	27,98	24,80	0	0	Out
99	male	1	2013	21	27,98	32,90	2	0	Out
99	male	3	2013	21	27,98	36,00	2	0	Out
99	male	5	2013	21	27,98	32,00	2	0	Out

99	male	10	2013	21	27,98	32,00	2	0	Out
99	male	12	2013	21	27,98	40,00	2	0	Out
99	male	5	2014	22	27,98	39,10	2	0	Out
99	male	12	2014	22	27,98	30,80	0	0	Out
100	male	6	2012	25	27,25	36,00	0	0	Out
100	male	1	2013	26	27,25	28,30	1	0	Out
100	male	5	2013	26	27,25	49,00	1	1	Out
100	male	11	2013	26	27,25	26,00	0	0	Out
100	male	8	2014	27	27,25	67,40	0	0	Out
100	male	2	2015	28	27,25	47,90	2	0	Out
101	male	4	2010	21	23,13	28,77	1	0	Out
101	male	10	2010	21	23,08	23,37	0	0	Out
101	male	2	2011	22	22,82	38,60	1	1	Out
101	male	3	2011	22	22,82	23,80	0	0	Out
101	male	2	2012	23	22,82	54,80	1	1	Out
101	male	2	2013	24	22,82	48,00	1	1	Out
101	male	4	2013	24	22,82	39,00	1	1	Out
101	male	12	2013	24	22,82	37,00	0	0	Out
101	male	6	2014	25	22,82	32,20	0	0	Out
101	male	11	2014	25	22,82	27,80	0	0	Out
101	male	12	2014	25	22,82	24,10	0	0	Out
102	male	10	2010	21	21,71	22,15	0	0	Out
102	male	6	2012	23	21,71	39,40	0	1	Out
102	male	1	2013	24	21,71	27,50	1	0	Out
102	male	4	2013	24	21,71	29,00	1	1	Out
102	male	10	2013	24	21,71	24,00	0	0	Out
102	male	12	2013	24	21,71	34,00	2	0	Out
102	male	8	2014	25	21,71	43,10	0	0	Out
102	male	11	2014	25	21,71	31,80	0	0	Out
102	male	12	2014	25	21,71	36,70	2	0	Out
102	male	1	2015	26	21,71	43,00	1	2	Out
103	male	1	2010	24	22,98	20,94	0	0	Out
103	male	7	2010	24	22,88	25,49	0	0	Out
103	male	11	2010	24	22,88	19,44	0	0	Out
103	male	6	2011	25	22,88	44,60	0	1	Out
103	male	6	2012	26	22,88	41,30	0	1	Out
103	male	7	2012	26	22,88	38,40	0	1	Out
103	male	1	2013	27	22,88	26,90	1	0	Out
103	male	5	2013	27	22,88	44,00	1	1	Out
103	male	8	2014	28	22,88	48,50	0	0	Out
104	male	10	2014	21	21,42	48,60	0	0	Out

105	male	12	2009	23	21,71	19,99	0	0	Out
105	male	3	2010	25	21,71	37,70	1	1	Out
105	male	4	2010	25	21,71	49,10	1	1	Out
105	male	6	2010	24	21,86	26,15	0	1	Out
105	male	6	2010	25	21,71	37,40	0	1	Out
105	male	7	2010	24	21,74	27,12	0	1	Out
105	male	8	2010	24	21,71	29,85	0	0	Out
105	male	10	2010	24	21,71	20,95	0	0	Out
105	male	2	2011	25	21,71	22,40	0	0	Out
105	male	10	2011	26	21,71	19,60	0	0	Out
105	male	6	2012	27	21,71	37,00	0	1	Out
105	male	7	2012	27	21,71	35,80	0	1	Out
105	male	1	2013	28	21,71	22,90	0	0	Out
105	male	2	2013	28	21,71	21,00	0	0	Out
105	male	5	2013	28	21,71	40,00	1	1	Out
105	male	10	2013	28	21,71	41,00	2	0	Out
105	male	10	2013	28	21,71	19,00	0	0	Out
105	male	11	2013	28	21,71	42,00	2	0	Out
105	male	5	2014	29	21,71	37,00	2	0	Out
105	male	10	2014	29	21,71	34,30	0	0	Out
106	male	3	2012	24	22,71	58,70	1	1	Out
106	male	4	2012	24	22,71	51,70	1	1	Out
106	male	11	2012	24	22,71	45,30	0	0	Out
107	male	6	2012	24	22,71	36,70	0	0	Out
108	male	4	2013	19	22,63	13,00	0	0	Out
108	male	12	2013	19	22,63	23,00	0	0	Out
109	female	1	2011	26	17,96	74,00	1	0	Out
109	female	5	2011	26	17,78	48,50	1	0	Out
110	female	1	2013	21	23,97	24,40	0	0	Out
111	female	6	2010	23	18,82	25,08	0	0	Out
112	female	1	2013	28	17,98	40,60	2	0	Out
112	male	4	2013	28	17,98	22,00	0	0	Out
112	female	2	2015	30	17,98	28,70	0	0	Out
113	male	8	2014	28	22,54	25,20	0	0	Out
114	male	3	2011	24	20,42	57,80	1	1	Out
114	male	6	2011	24	20,42	42,00	0	1	Out
114	male	8	2011	24	20,42	45,30	0	1	Out
114	male	1	2012	25	20,42	65,20	1	0	Out
114	male	3	2013	26	20,42	57,00	1	1	Out
114	male	10	2013	26	20,42	29,00	0	0	Out
114	male	12	2013	26	20,42	62,00	1	0	Out

115	female	4	2014	28	19,94	26,10	0	0	Out
115	female	10	2014	28	19,94	38,40	0	0	Out
116	female	7	2011	25	19,33	21,90	0	0	Out
116	female	5	2012	26	19,33	44,10	1	1	Out
117	female	4	2014	24	19,95	49,70	1	0	Out
117	female	11	2014	24	19,95	30,30	0	0	Out
117	female	11	2014	24	19,95	33,50	0	0	Out
117	female	12	2014	24	19,95	42,80	2	0	Out
117	female	1	2015	25	19,95	67,80	1	2	Out
118	male	2	2013	21	24,96	34,00	0	0	Out
118	male	1	2014	22	24,96	36,00	2	0	Out
118	male	11	2014	22	24,96	24,00	0	0	Out
119	female	8	2011	35	24,34	33,00	0	0	Out
119	female	3	2013	37	24,34	12,00	0	0	Out
120	male	12	2009	28	33,88	26,07	0	0	Out
120	male	4	2010	29	38,11	34,79	1	1	Out
120	male	6	2010	29	33,64	28,55	0	0	Out
120	male	11	2010	29	33,64	19,13	0	0	Out
120	male	1	2011	30	33,64	19,20	0	0	Out
120	male	4	2011	30	33,64	35,40	1	0	Out
120	male	6	2011	30	34,60	30,10	0	0	Out
120	male	10	2011	30	33,33	30,70	2	0	Out
120	male	4	2012	31	33,33	48,50	1	0	Out
120	male	5	2012	31	33,33	37,50	1	0	Out
120	male	3	2013	32	33,33	13,00	0	0	Out
120	male	1	2014	33	33,33	27,00	0	0	Out
120	male	5	2014	33	33,33	24,70	0	0	Out
120	male	10	2014	33	33,33	16,90	0	0	Out
120	male	12	2014	33	33,33	22,50	0	0	Out
121	female	2	2015	21	34,15	12,10	0	0	Out
122	male	4	2011	28	35,81	27,90	0	1	Out
122	male	8	2011	28	35,81	24,80	0	0	Out
122	male	2	2012	29	35,81	62,30	1	1	Out
122	male	3	2012	29	35,81	57,40	1	1	Out
122	male	6	2012	29	35,81	49,20	0	0	Out
122	male	5	2013	30	35,81	29,00	0	1	Out
122	male	2	2014	31	35,81	36,00	1	0	Out
123	male	11	2009	27	24,76	36,63	0	0	Out
123	male	4	2010	28	23,69	35,62	1	0	Out
123	male	10	2010	28	24,78	28,47	0	0	Out
123	male	2	2011	29	25,08	32,30	1	0	Out

123	male	4	2011	29	25,08	29,10	1	0	Out
123	male	7	2011	29	25,08	38,30	0	1	Out
123	male	2	2012	30	25,08	45,70	1	0	Out
123	male	11	2012	30	25,08	27,00	0	0	Out
123	male	2	2013	31	25,08	45,00	1	0	Out
123	male	7	2013	31	25,08	36,00	0	0	Out
123	male	12	2013	31	25,08	22,00	0	0	Out
123	male	7	2014	32	25,08	30,20	0	0	Out
124	female	10	2012	18	21,71	22,80	0	0	Out
124	female	1	2013	19	21,71	35,40	2	0	Out
124	female	9	2013	19	21,71	41,00	2	0	Out
124	female	12	2013	19	21,71	25,00	0	0	Out
125	male	5	2010	26	22,98	57,90	1	1	Out
125	male	7	2011	27	22,98	46,30	0	1	Out
125	male	11	2011	27	22,98	47,80	2	0	Out
125	male	2	2012	28	22,98	58,10	2	0	Out
125	male	11	2012	28	22,98	63,00	2	0	Out
125	male	4	2013	29	22,98	65,00	1	0	Out
125	male	5	2014	30	22,98	53,40	1	0	Out
125	male	7	2014	30	22,98	49,10	2	0	Out
125	male	2	2015	31	22,98	36,60	2	0	Out
126	male	11	2012	17	20,87	17,50	0	0	Out
127	female	3	2012	24	20,77	71,20	1	0	Out
127	female	4	2012	24	20,77	55,70	1	0	Out
127	female	12	2014	26	20,77	31,90	2	0	Out
128	male	3	2013	19	23,30	19,00	0	0	Out
129	male	8	2011	23	24,21	43,60	0	0	Out
129	male	7	2012	24	24,21	35,20	0	0	Out
130	female	11	2014	22	20,44	24,90	0	0	Out
131	female	6	2012	22	19,95	37,20	0	0	Out
131	female	7	2014	24	19,95	48,60	2	0	Out
132	male	8	2010	23	34,18	29,80	0	0	Out
133	male	2	2010	21	22,76	25,49	0	0	Out
134	male	12	2009	21	23,95	23,78	0	0	Out
134	male	3	2010	22	23,95	19,40	0	0	Out
134	male	4	2010	22	23,93	42,79	1	0	Out
134	male	6	2010	22	23,96	36,11	0	1	Out
134	male	7	2010	22	24,73	30,52	0	1	Out
134	male	10	2010	22	24,60	32,12	0	0	Out
134	male	11	2010	22	25,51	32,74	0	0	Out
134	male	1	2011	23	25,51	37,90	1	0	Out

134	male	3	2011	23	25,51	28,70	0	1	Out
134	male	5	2011	23	23,45	46,40	1	1	Out
134	male	11	2011	23	23,95	49,20	0	0	Out
134	male	2	2012	24	23,95	39,50	2	0	Out
134	male	3	2012	24	23,95	27,30	2	0	Out
134	male	4	2012	24	23,95	27,30	2	0	Out
134	male	4	2012	24	23,95	41,10	1	0	Out
134	male	7	2012	24	23,95	40,20	0	0	Out
134	male	11	2012	24	23,95	32,30	2	0	Out
134	male	1	2013	25	23,95	41,00	1	0	Out
134	male	2	2013	25	23,95	63,00	1	0	Out
134	male	3	2013	25	23,95	56,00	1	0	Out
134	male	4	2013	25	23,95	44,00	1	1	Out
134	male	7	2013	25	23,95	44,00	0	1	Out
134	male	11	2013	25	23,95	30,00	0	0	Out
134	male	3	2014	26	23,95	19,00	0	0	Out
134	male	10	2014	26	23,95	39,50	0	0	Out
135	male	1	2013	24	17,41	11,20	0	0	Out
136	male	11	2010	22	21,61	18,11	0	0	Out
136	male	1	2011	23	21,61	30,90	1	0	Out
136	male	5	2011	23	20,83	28,30	0	1	Out
136	male	11	2012	24	20,83	28,70	0	0	Out
136	male	7	2014	26	20,83	44,80	0	0	Out
136	male	12	2014	26	20,83	25,60	0	0	Out
137	male	11	2010	26	21,65	18,86	0	0	Out
137	male	2	2011	27	21,65	42,70	1	0	Out
137	male	3	2011	27	21,65	27,30	0	0	Out
137	male	5	2012	28	21,65	51,70	1	1	Out
137	male	2	2013	29	21,65	38,00	0	0	Out
137	male	11	2013	29	21,65	29,00	0	0	Out
138	male	11	2014	20	26,88	17,50	0	0	Out
139	male	2	2013	24	30,69	28,00	0	0	Out
139	male	7	2013	24	30,69	29,00	0	0	Out
140	male	2	2013	19	37,09	17,00	0	0	Out
140	male	12	2014	20	37,09	17,80	0	0	Out
141	female	6	2010	19	32,49	27,12	0	0	Out
142	female	10	2014	21	20,06	29,90	0	0	Out
143	male	5	2013	21	22,69	42,00	1	1	Out
143	male	11	2013	21	22,69	15,00	0	0	Out
143	male	1	2014	22	22,69	32,00	2	0	Out
143	male	5	2014	22	22,69	31,30	1	0	Out

143	male	10	2014	22	22,69	23,20	0	0	Out
144	male	11	2010	21	20,16	18,90	0	0	Out
144	male	1	2013	24	20,16	39,70	1	0	Out
144	male	5	2013	24	20,16	43,00	1	1	Out
144	male	7	2013	24	20,16	38,00	0	1	Out
144	male	4	2014	25	20,16	41,30	1	0	Out
144	male	10	2014	25	20,16	32,00	0	0	Out
144	male	12	2014	25	20,16	38,10	2	0	Out
144	male	1	2015	26	20,16	54,00	1	2	Out
145	female	6	2011	20	20,92	41,70	0	1	Out
145	female	7	2012	21	20,92	41,00	0	1	Out
145	female	1	2013	22	20,92	31,10	1	0	Out
145	female	7	2013	22	20,92	32,00	0	1	Out
145	female	11	2013	22	20,92	22,00	0	0	Out
146	male	6	2012	21	28,77	15,80	0	0	Out
146	male	12	2012	21	28,77	14,20	0	0	Out
147	male	11	2012	24	18,17	19,90	0	0	Out
147	male	1	2013	25	18,17	26,70	2	0	Out
147	male	3	2013	25	18,17	48,00	2	0	Out
147	male	4	2013	25	18,17	40,00	2	0	Out
147	male	5	2013	25	18,17	37,00	2	0	Out
147	male	11	2013	25	18,17	30,00	0	0	Out
147	male	12	2013	25	18,17	36,00	2	0	Out
147	male	4	2014	26	18,17	21,10	0	0	Out
147	male	5	2014	26	18,17	42,00	2	0	Out
148	male	6	2012	23	19,38	35,00	1	0	Out
148	male	1	2013	24	19,38	25,50	0	0	Out
149	male	11	2010	23	21,33	23,35	0	0	Out
150	male	11	2010	25	21,11	19,72	0	0	Out
150	male	2	2011	26	21,14	25,00	0	0	Out
150	male	4	2011	26	21,14	36,70	1	0	Out
150	male	6	2011	26	20,99	35,70	0	1	Out
150	male	4	2012	27	20,99	32,80	1	1	Out
150	male	5	2012	27	20,99	44,80	0	1	Out
150	male	12	2012	27	20,99	39,00	0	0	Out
150	male	4	2013	28	20,99	34,00	2	0	Out
150	male	5	2013	28	20,99	30,00	2	0	Out
150	male	7	2013	28	20,99	36,00	0	0	Out
150	male	1	2014	29	20,99	29,00	0	0	Out
150	male	6	2014	29	20,99	29,50	0	0	Out
150	male	11	2014	29	20,99	31,50	0	0	Out

151	male	1	2010	22	24,91	22,87	0	0	Out
151	male	7	2010	22	24,91	28,18	0	0	Out
151	male	11	2010	22	24,91	22,16	0	0	Out
151	male	2	2011	23	25,71	25,40	0	0	Out
151	male	4	2011	23	25,71	39,00	1	0	Out
151	male	6	2011	23	25,71	43,90	0	1	Out
151	male	3	2012	24	25,71	14,20	0	0	Out
152	male	11	2012	18	19,43	26,60	0	0	Out
153	male	8	2014	22	24,81	57,00	0	0	Out
154	male	5	2012	23	23,63	58,70	1	1	Out
154	male	12	2012	23	23,63	33,40	0	0	Out
154	male	11	2014	25	23,63	22,30	0	0	Out
155	female	11	2010	27	23,42	24,44	0	0	Out
155	female	5	2011	27	22,41	27,00	0	0	Out
156	female	11	2009	26	21,05	44,57	0	0	Out
156	female	11	2010	27	21,96	37,16	0	0	Out
156	female	5	2011	27	22,05	26,20	0	0	Out
156	female	11	2011	27	22,09	44,00	0	0	Out
156	female	5	2012	28	22,09	56,40	0	1	Out
157	male	7	2011	30	21,74	42,70	0	1	Out
157	male	5	2012	31	21,74	49,40	1	1	Out
157	male	2	2013	33	21,74	29,00	0	0	Out
158	male	12	2012	22	28,91	28,70	0	0	Out
159	female	11	2010	25	17,95	13,79	0	0	Out
159	female	2	2011	26	17,88	48,60	1	0	Out
159	female	6	2011	26	17,88	29,10	0	1	Out
159	female	4	2012	27	17,88	22,20	0	0	Out
159	female	2	2013	28	17,88	25,00	0	1	Out
159	female	3	2013	28	17,88	25,00	0	1	Out
159	female	12	2014	29	17,88	17,70	0	0	Out
159	female	2	2015	30	17,88	32,90	1	0	Out
160	female	12	2009	21	20,90	35,64	0	0	Out
160	female	5	2010	22	20,17	42,12	1	0	Out
160	female	6	2010	22	20,17	26,82	0	0	Out
160	female	11	2010	22	20,63	26,59	0	0	Out
160	female	5	2011	23	20,37	33,90	1	0	Out
160	female	12	2011	23	19,78	30,40	0	0	Out
161	female	5	2010	22	19,95	29,70	1	0	Out
161	female	6	2010	22	19,61	23,62	0	1	Out
161	female	7	2010	22	19,87	24,63	0	0	Out
161	female	9	2010	22	19,87	21,79	0	0	Out

161	female	10	2010	22	19,87	18,55	0	0	Out
161	female	12	2010	22	20,14	17,41	0	0	Out
161	female	1	2011	23	20,14	18,12	0	0	Out
161	female	2	2011	23	20,14	37,70	1	0	Out
161	female	4	2011	23	20,32	35,10	1	1	Out
161	female	7	2011	23	20,85	39,10	0	1	Out
161	female	1	2012	24	20,85	27,60	0	0	Out
161	female	4	2012	24	20,85	45,20	1	1	Out
161	female	5	2012	24	20,85	37,70	1	1	Out
161	female	7	2012	24	20,85	31,10	1	1	Out
161	female	11	2014	24	20,85	24,00	0	0	Out
162	female	11	2010	30	29,57	36,24	0	0	Out
163	male	2	2015	22	28,98	12,20	0	0	Out
164	female	11	2014	22	30,87	17,70	0	0	Out
165	female	12	2009	22	21,40	31,03	0	0	Out
165	female	5	2010	23	20,52	28,56	1	0	Out
165	female	6	2010	23	20,52	29,07	0	1	Out
165	female	7	2010	23	20,52	25,28	0	0	Out
165	female	11	2010	23	20,52	36,16	2	0	Out
165	female	1	2011	24	20,82	33,47	2	0	Out
165	female	2	2011	24	20,82	51,70	1	0	Out
165	female	4	2011	24	21,19	40,30	1	1	Out
165	female	5	2011	24	21,56	35,50	1	1	Out
165	female	7	2011	24	21,56	34,00	0	1	Out
165	female	1	2012	25	21,56	40,40	1	0	Out
165	female	5	2012	25	21,56	49,80	1	1	Out
165	female	7	2012	25	21,56	44,80	0	1	Out
165	female	12	2012	25	21,56	36,70	2	0	Out
165	female	1	2013	26	21,56	25,50	0	0	Out
165	female	6	2013	26	21,56	41,00	0	1	Out
165	female	1	2014	27	21,56	28,00	0	0	Out
166	female	12	2009	29	19,84	29,86	0	0	Out
166	female	1	2010	30	19,84	35,01	0	0	Out
166	female	11	2010	30	19,84	21,97	0	0	Out
166	female	8	2011	31	20,34	39,10	0	0	Out
166	female	10	2011	31	20,34	34,80	0	0	Out
167	male	11	2010	23	25,65	18,37	0	0	Out
167	male	6	2012	25	25,65	41,10	0	0	Out
167	male	12	2012	25	25,65	33,30	2	0	Out
167	male	11	2014	27	25,65	28,00	0	0	Out
168	male	7	2010	20	34,90	17,29	0	0	Out

169	female	11	2010	28	19,74	18,52	0	0	Out
169	female	5	2011	28	19,49	48,90	1	0	Out
170	female	10	2009	28	20,36	36,40	1	0	Out
170	female	11	2010	29	20,40	30,55	0	0	Out
170	female	1	2011	30	20,41	48,60	1	0	Out
170	female	5	2011	30	20,41	47,00	1	1	Out
170	female	10	2011	30	20,40	48,80	0	0	Out
170	female	4	2012	31	20,40	58,60	1	1	Out
170	female	10	2012	31	20,40	52,40	0	0	Out
171	male	11	2014	21	31,80	29,60	0	0	Out
171	male	2	2015	22	31,80	37,20	2	0	Out
172	female	11	2012	32	19,09	24,20	0	0	Out
173	male	2	2011	20	20,19	41,70	0	0	Out
174	male	11	2009	22	20,35	30,85	0	0	Out
175	male	1	2011	21	19,12	20,60	0	0	Out
176	female	11	2010	16	21,01	17,00	0	0	Out
176	female	4	2011	17	20,31	17,00	0	0	Out
177	female	1	2013	23	19,22	20,40	0	0	Out
178	female	2	2012	32	20,00	21,20	0	0	Out
179	female	11	2009	29	20,00	63,87	1	0	Out
179	female	5	2010	30	19,72	21,87	0	0	Out
179	female	6	2010	30	20,07	45,70	0	1	Out
179	female	8	2010	30	19,72	43,93	0	1	Out
179	female	2	2011	31	19,72	59,20	1	0	Out
179	female	4	2011	31	19,72	38,20	1	1	Out
179	female	5	2012	32	19,72	37,80	1	0	Out
180	male	12	2013	30	19,72	38,00	2	0	Out
180	male	4	2014	31	19,72	31,30	2	0	Out
180	male	8	2014	31	19,72	44,00	0	0	Out
180	male	10	2014	31	19,72	22,70	0	0	Out
180	male	12	2014	31	19,72	26,80	0	0	Out
181	male	11	2010	23	20,37	23,51	0	0	Out
181	male	2	2011	24	20,65	43,80	1	0	Out
181	male	5	2011	24	20,74	29,80	0	1	Out
181	male	9	2011	24	21,50	40,90	2	0	Out
181	male	11	2011	24	21,50	35,70	2	0	Out
181	male	4	2013	26	21,50	44,00	1	0	Out
181	male	1	2014	27	21,50	35,00	1	0	Out
181	male	6	2014	27	21,50	33,70	0	0	Out
182	female	4	2010	25	20,25	36,85	1	0	Out
183	male	11	2012	21	19,72	23,90	0	0	Out

183	male	4	2013	22	19,72	18,00	0	0	Out
184	male	3	2014	21	22,59	43,00	2	0	Out
185	male	10	2013	22	22,35	22,00	0	0	Out
185	male	1	2014	23	22,35	38,00	2	0	Out
185	male	10	2014	23	22,35	26,70	0	0	Out
185	male	12	2014	23	22,35	38,10	2	0	Out
186	female	9	2010	24	22,92	27,41	0	0	Out
187	male	3	2012	25	23,46	18,80	0	0	Out
188	female	1	2013	22	31,81	24,90	0	0	Out
189	male	12	2009	24	21,45	29,68	0	0	Out
189	male	6	2010	25	21,39	35,30	0	0	Out
189	male	5	2011	26	21,55	46,80	2	0	Out
189	male	1	2013	28	21,55	47,60	2	0	Out
189	male	7	2013	28	21,55	41,00	0	0	Out
190	male	11	2012	24	21,16	20,50	0	0	Out
190	male	5	2013	25	21,16	24,00	0	0	Out
191	male	5	2012	25	25,88	44,50	1	0	Out
192	female	1	2014	21	21,78	23,00	0	0	Out
193	male	12	2012	22	22,25	12,70	0	0	Out
193	male	12	2014	22	22,25	14,70	0	0	Out
194	male	1	2010	32	20,50	20,71	0	0	Out
194	male	2	2010	32	20,50	17,45	0	0	Out
194	male	7	2010	32	20,50	22,79	0	0	Out
194	male	8	2010	32	19,89	35,92	2	0	Out
194	male	1	2011	33	20,27	22,30	0	0	Out
194	male	4	2011	33	21,06	35,00	1	1	Out
194	male	8	2011	33	21,06	38,50	0	0	Out
194	male	11	2011	33	21,06	24,60	0	0	Out
194	male	11	2012	34	21,06	33,00	2	0	Out
194	male	6	2013	35	21,06	26,00	0	0	Out
194	male	11	2014	36	21,06	24,10	0	0	Out
195	male	11	2014	21	20,65	22,80	0	0	Out
196	male	4	2013	20	23,06	14,00	0	0	Out
197	male	5	2014	23	24,63	33,90	0	0	Out
198	female	6	2012	23	18,13	53,50	1	1	Out
198	female	11	2012	23	18,13	39,40	0	0	Out
198	female	6	2013	24	18,13	31,00	0	0	Out
199	male	6	2012	30	20,39	23,30	0	0	Out
199	male	5	2013	31	20,39	25,00	0	0	Out
199	male	12	2014	32	20,39	19,40	0	0	Out
200	female	12	2009	22	20,01	26,82	0	0	Out

201	female	11	2012	26	20,20	22,20	0	0	Out
201	female	4	2013	27	20,20	35,00	1	0	Out
202	male	4	2011	28	21,07	63,90	1	0	Out
202	male	8	2011	28	20,43	62,10	0	0	Out
202	male	3	2012	29	20,43	76,30	1	0	Out
203	male	10	2013	22	33,28	27,00	0	0	Out
203	male	12	2013	22	33,28	26,00	0	0	Out
203	male	1	2014	23	33,28	48,00	2	0	Out
203	male	12	2014	23	33,28	30,60	0	0	Out
204	male	11	2010	24	22,02	14,33	0	0	Out
204	male	5	2011	25	21,95	24,30	0	0	Out
205	female	4	2012	20	21,05	43,70	1	0	Out
205	female	7	2012	20	21,05	37,10	0	1	Out
205	female	12	2012	20	21,05	27,70	1	0	Out
205	female	1	2013	21	21,05	44,70	1	0	Out
205	female	2	2013	21	21,05	48,00	1	1	Out
205	female	4	2013	21	21,05	55,00	1	1	Out
205	female	7	2013	21	21,05	31,00	0	0	Out
205	female	6	2014	22	21,05	57,50	2	0	Out
205	female	8	2014	22	21,05	77,30	0	0	Out
205	female	10	2014	22	21,05	35,60	0	0	Out
205	female	11	2014	22	21,05	24,50	0	0	Out
205	female	12	2014	22	21,05	50,90	2	0	Out
205	female	1	2015	23	21,05	108,20	1	2	Out
206	female	1	2013	26	21,07	42,90	2	0	Out
206	female	4	2013	26	21,07	37,00	2	0	Out
206	female	9	2013	26	21,07	43,00	2	0	Out
207	male	7	2010	24	23,81	28,30	0	0	Out
207	male	11	2010	24	23,81	20,45	0	0	Out
207	male	5	2011	25	23,11	27,70	0	0	Out
208	male	11	2009	20	18,72	63,87	1	0	Out
208	male	6	2010	21	18,72	27,45	0	0	Out
208	male	11	2010	21	18,72	15,43	0	0	Out
208	male	12	2012	23	18,72	28,60	0	0	Out
208	male	12	2014	25	18,72	25,70	0	0	Out
209	female	3	2014	33	18,94	23,00	0	0	Out
209	female	11	2014	33	18,94	27,40	0	0	Out
210	female	11	2009	28	18,94	27,20	0	0	Out
210	female	3	2010	29	19,27	27,41	1	0	Out
210	female	5	2010	29	19,27	21,98	0	0	Out
210	female	6	2010	29	18,51	32,83	0	0	Out

210	female	7	2010	29	18,35	30,48	0	0	Out
210	female	9	2010	29	18,87	21,50	0	0	Out
210	female	10	2010	29	18,87	35,49	0	0	Out
210	female	12	2010	29	18,87	24,92	0	0	Out
210	female	1	2011	30	19,59	25,41	0	0	Out
210	female	2	2011	30	19,59	24,90	0	0	Out
210	female	3	2011	30	19,59	40,00	1	0	Out
210	female	4	2011	30	19,59	36,70	0	1	Out
210	female	10	2011	30	19,59	39,40	2	0	Out
210	female	11	2011	30	19,59	46,60	2	0	Out
210	female	1	2012	31	19,59	45,30	2	0	Out
210	female	3	2012	31	19,59	39,10	2	0	Out
210	female	4	2012	31	19,59	39,10	2	0	Out
211	female	1	2010	26	23,84	35,98	0	0	Out
211	female	7	2010	26	23,61	32,78	0	0	Out
211	female	1	2011	27	23,78	59,20	1	0	Out
211	female	12	2011	27	25,23	36,50	0	0	Out
211	female	7	2012	28	25,23	44,10	0	0	Out
211	female	2	2015	31	25,23	48,10	1	0	Out
212	male	6	2011	24	29,42	33,50	0	0	Out
212	male	2	2012	25	29,42	58,70	1	0	Out
212	male	3	2012	25	29,42	57,30	1	1	Out
212	male	6	2012	25	29,42	40,00	0	1	Out
212	male	7	2012	25	29,42	44,10	0	1	Out
212	male	11	2012	25	29,42	34,90	0	0	Out
212	male	6	2013	26	29,42	36,00	0	0	Out
212	male	9	2013	26	29,42	29,00	0	0	Out
212	male	12	2013	26	29,42	35,00	2	0	Out
212	male	11	2014	27	29,42	31,90	0	0	Out
213	female	1	2012	23	18,26	30,60	0	0	Out
213	female	11	2012	23	18,26	31,60	0	0	Out
213	female	11	2014	25	18,26	29,30	0	0	Out
214	male	1	2013	19	22,50	22,00	0	0	Out
215	male	10	2013	18	32,62	22,00	0	0	Out
216	female	4	2010	21	19,20	34,55	1	0	Out
216	female	7	2010	21	18,50	26,12	0	1	Out
216	female	1	2011	22	18,50	24,66	0	0	Out
216	female	2	2011	22	19,16	62,50	1	0	Out
216	female	5	2011	22	19,98	51,70	0	1	Out
216	female	7	2011	22	19,98	47,00	0	1	Out
216	female	11	2011	22	18,87	61,30	0	0	Out

216	female	4	2012	23	18,87	95,70	1	1	Out
216	female	1	2013	24	18,87	54,70	1	0	Out
216	female	4	2013	24	18,87	30,00	0	1	Out
216	female	1	2014	25	18,87	38,00	0	0	Out
216	female	2	2015	26	18,87	46,00	2	0	Out
217	male	7	2010	26	23,46	34,08	0	0	Out
217	male	11	2010	26	23,46	24,32	0	0	Out
217	male	11	2011	26	22,15	34,30	0	0	Out
217	male	2	2012	27	22,15	62,70	1	0	Out
217	male	6	2012	27	22,15	51,30	0	1	Out
217	male	11	2012	27	22,15	36,60	0	0	Out
217	male	3	2013	28	22,15	56,00	1	0	Out
217	male	10	2013	28	22,15	29,00	0	0	Out
218	female	3	2012	24	20,76	56,00	1	0	Out
219	male	11	2009	28	22,32	4,20	0	0	Out
219	male	4	2010	29	23,10	28,00	1	0	Out
220	male	12	2014	23	24,27	28,60	0	0	Out
221	male	2	2011	22	24,21	26,10	0	0	Out
222	female	10	2010	38	30,02	19,51	0	0	Out
222	female	1	2011	29	30,02	16,50	0	0	Out
222	female	5	2011	29	30,41	21,20	0	0	Out
222	female	10	2011	29	29,86	20,50	0	0	Out
222	female	3	2012	30	29,86	23,60	0	0	Out
222	female	1	2013	31	29,86	19,80	0	0	Out
222	female	5	2013	31	29,86	25,00	0	0	Out
223	female	9	2013	21	32,99	38,00	2	0	Out
223	male	3	2014	22	32,99	26,00	0	0	Out
223	male	10	2014	22	32,99	28,70	0	0	Out
223	male	11	2014	22	32,99	29,40	0	0	Out
224	female	12	2014	19	21,75	24,90	0	0	Out
224	female	2	2015	20	21,75	32,90	2	0	Out
225	female	5	2013	20	35,66	14,00	0	0	Out
225	female	10	2013	20	35,66	19,00	0	0	Out
225	female	1	2014	21	35,66	23,00	0	0	Out
225	female	12	2014	21	35,66	18,70	0	0	Out
226	female	1	2010	25	28,73	48,71	1	0	Out
226	female	11	2010	25	30,64	29,81	0	0	Out
226	female	4	2011	26	29,70	43,30	1	1	Out
226	female	5	2011	26	29,70	37,30	0	1	Out
226	female	11	2011	26	29,70	42,20	0	0	Out
226	female	1	2013	28	29,70	35,30	1	0	Out

226	female	2	2013	28	29,70	47,00	1	1	Out
226	female	4	2013	28	29,70	46,00	1	1	Out
226	female	11	2013	28	29,70	37,00	0	1	Out
226	female	10	2014	29	29,70	45,00	0	0	Out
227	male	7	2010	22	21,97	24,93	0	0	Out
227	male	2	2011	23	21,97	21,60	0	0	Out
227	male	8	2011	23	21,97	28,30	0	0	Out
227	male	1	2012	24	21,97	33,90	1	0	Out
227	male	7	2012	24	21,97	47,10	0	0	Out
227	male	3	2013	25	21,97	27,00	0	0	Out
228	male	7	2010	22	21,97	24,93	0	0	Out
229	female	4	2013	19	19,71	19,00	0	0	Out
229	female	6	2013	19	19,71	23,00	0	0	Out
229	female	8	2014	20	19,71	33,30	0	0	Out
229	female	2	2015	21	19,71	45,30	0	0	Out
230	male	1	2010	21	23,77	26,00	0	0	Out
230	male	11	2010	21	24,23	17,34	0	0	Out
230	male	6	2011	22	23,95	61,20	1	1	Out
230	male	6	2012	23	23,95	60,10	1	1	Out
230	male	7	2012	23	23,95	60,60	0	0	Out
230	male	10	2014	25	23,95	29,60	0	0	Out
231	female	7	2010	28	31,46	21,21	0	0	Out
232	male	12	2014	21	23,71	40,00	0	0	Out
233	male	4	2012	23	20,45	56,60	1	0	Out
233	male	11	2012	23	20,45	43,40	1	0	Out
234	male	8	2010	23	31,86	36,91	0	1	Out
234	male	3	2011	24	31,86	24,30	0	0	Out
234	male	2	2012	25	31,86	39,40	1	0	Out
235	female	6	2010	24	19,21	31,53	0	0	Out
235	female	8	2010	24	19,21	36,91	0	0	Out
235	female	1	2011	25	19,38	28,10	0	0	Out
235	female	2	2011	25	19,72	99,40	1	0	Out
235	female	4	2011	25	19,72	43,20	0	1	Out
235	female	5	2011	25	19,72	38,70	0	1	Out
235	female	7	2011	25	19,72	52,50	0	0	Out
236	male	11	2010	19	24,25	26,17	0	0	Out
236	male	2	2011	20	24,54	35,70	2	0	Out
236	male	7	2011	20	25,11	58,40	0	1	Out
236	male	11	2012	21	25,11	36,80	2	0	Out
236	male	1	2013	22	25,11	29,30	2	0	Out
236	male	7	2013	23	25,11	41,00	0	1	Out

236	male	10	2014	24	25,11	32,60	0	0	Out
237	female	1	2010	30	20,82	32,98	0	0	Out
237	female	8	2010	30	21,49	33,98	0	0	Out
238	male	2	2010	34	37,95	23,04	0	0	Out
238	male	7	2010	34	37,18	18,95	0	0	Out
238	male	11	2010	34	33,60	18,42	0	0	Out
238	male	1	2011	35	33,46	25,60	0	0	Out
238	male	1	2012	36	33,46	16,20	0	0	Out
239	male	6	2012	22	19,63	46,70	0	1	Out
239	male	12	2013	23	19,63	31,00	0	0	Out
239	male	5	2014	24	19,63	47,20	1	0	Out
240	m	12	2011	16	12,50	24,45	0	0	In
241	m	7	2014	32	38,90	29,45	1	0	In
241	m	2	2015	33	20,90	29,45	0	0	In
242	k	12	2011	15	24,60	22,94	0	0	In
243	k	5	2009	24	21,90	39,67	0	0	In
243	k	10	2009	24	23,10	39,67	0	0	In
243	k	5	2010	25	25,12	39,67	0	0	In
243	k	7	2010	25	25,63	39,67	0	0	In
243	k	5	2011	26	30,80	39,67	0	0	In
243	k	9	2011	26	33,20	39,67	0	0	In
243	k	12	2011	26	29,90	39,67	0	0	In
243	k	5	2012	27	40,10	39,67	0	0	In
243	k	12	2012	27	25,20	39,67	0	0	In
244	k	5	2009	18	32,16	23,11	0	0	In
244	k	10	2009	18	33,66	23,11	0	0	In
245	m	4	2012	25	16,30	24,49	0	0	In
245	m	4	2013	26	18,00	24,49	0	0	In
246	k	4	2012	26	36,60	21,86	0	0	In
247	k	4	2012	32	24,60	20,23	0	0	In
247	k	4	2014	34	21,90	20,23	0	0	In
248	m	10	2009	27	22,94	25,98	0	0	In
248	m	1	2010	28	19,63	25,98	0	0	In
248	m	1	2012	30	14,40	25,98	0	0	In
248	m	7	2014	32	27,70	25,98	1	0	In
248	m	2	2015	33	26,00	25,98	0	0	In
249	m	4	2013	22	22,00	24,74	0	0	In
249	m	5	2014	23	16,10	24,74	0	0	In
250	m	9	2011	27	19,60	31,13	0	0	In
251	m	7	2014	32	32,70	28,06	1	0	In
251	m	2	2015	33	15,50	28,06	0	0	In

252	m	5	2014	27	21,00	23,43	0	0	In
253	k	12	2011	19	34,40	21,45	0	0	In
254	m	6	2011	23	36,20	24,51	0	0	In
254	m	7	2014	26	41,80	24,51	1	0	In
254	m	2	2015	27	28,20	24,51	0	0	In
255	m	9	2011	24	17,00	33,41	0	0	In
256	m	7	2014	28	24,90	24,30	1	0	In
256	m	2	2015	29	12,20	24,30	0	0	In
257	m	9	2011	29	33,50	33,38	0	0	In
258	m	4	2012	22	13,80	24,98	0	0	In
258	m	4	2013	23	13,00	24,98	0	0	In
258	m	5	2014	24	26,90	24,98	0	0	In
259	k	4	2014	25	20,40	22,76	0	0	In
260	k	5	2010	21	26,94	21,08	0	0	In
261	k	5	2011	18	39,30	20,17	0	0	In
261	k	12	2011	18	20,80	20,17	0	0	In
261	k	5	2012	19	29,60	20,17	0	0	In
261	k	12	2012	19	26,80	20,17	0	0	In
262	m	4	2012	21	14,30	25,50	0	0	In
262	m	4	2013	22	15,00	25,50	0	0	In
262	m	5	2014	23	16,30	25,50	0	0	In
263	k	12	2012	23	22,60	19,88	0	0	In
264	k	5	2010	22	45,99	19,23	0	0	In
265	m	1	2010	28	20,02	29,59	0	0	In
266	k	5	2009	29	20,34	25,08	0	0	In
266	k	5	2011	31	27,50	25,08	0	0	In
267	m	12	2011	18	21,70	36,16	0	0	In
268	m	6	2011	29	24,10	25,00	0	0	In
268	m	7	2014	32	28,70	25,00	1	0	In
268	m	2	2015	33	27,20	25,00	0	0	In
269	k	12	2011	14	10,00	24,17	0	0	In
270	k	5	2011	22	31,20	21,36	0	0	In
271	k	5	2010	22	24,66	24,34	0	0	In
272	m	4	2013	22	17,00	21,00	0	0	In
273	m	4	2012	34	15,60	25,74	0	0	In
273	m	4	2013	35	14,00	25,74	0	0	In
273	m	5	2014	36	22,20	21,00	0	0	In
274	m	10	2009	25	16,49	25,23	0	0	In
274	m	1	2010	26	24,70	25,23	0	0	In
274	m	7	2014	30	25,70	25,23	1	0	In
274	m	2	2015	31	14,20	25,23	0	0	In

275	m	4	2012	25	26,60	23,96	0	0	In
275	m	4	2013	26	26,00	23,96	0	0	In
276	k	12	2011	19	20,40	22,81	0	0	In
277	m	10	2009	26	16,12	26,58	0	0	In
277	m	1	2010	27	14,87	26,58	0	0	In
277	m	6	2011	28	28,70	26,58	0	0	In
277	m	1	2012	29	16,00	26,58	0	0	In
278	m	10	2009	30	19,98	30,70	0	0	In
278	m	1	2010	31	15,16	30,70	0	0	In
278	m	6	2011	32	34,60	30,70	0	0	In
278	m	1	2012	33	24,00	30,70	0	0	In
279	m	10	2009	25	21,08	28,57	0	0	In
279	m	6	2011	27	20,50	28,57	0	0	In
279	m	1	2012	28	23,60	28,57	0	0	In
279	m	7	2014	30	33,70	28,57	1	0	In
279	m	2	2015	31	22,00	28,57	0	0	In
280	m	1	2010	28	11,68	26,26	0	0	In
280	m	6	2011	29	30,20	26,26	0	0	In
280	m	1	2012	30	20,80	26,26	0	0	In
281	k	12	2011	14	11,60	25,51	0	0	In
282	k	5	2010	26	33,01	18,72	0	0	In
282	k	4	2012	28	52,20	18,72	0	0	In
283	k	5	2010	22	21,18	20,01	0	0	In
283	k	4	2014	26	31,40	20,01	0	0	In
284	k	4	2014	24	24,20	22,59	0	0	In
285	k	5	2009	21	28,92	22,67	0	0	In
285	k	10	2009	21	27,00	22,67	0	0	In
285	k	5	2010	22	28,65	22,67	0	0	In
285	k	7	2010	22	28,80	22,67	0	0	In
285	k	5	2011	23	21,20	22,67	0	0	In
285	k	9	2011	23	25,30	22,67	0	0	In
285	k	5	2012	24	41,30	22,67	0	0	In
286	m	9	2011	24	26,50	33,31	0	0	In
287	k	5	2010	23	29,44	23,20	0	0	In
287	k	4	2012	25	39,50	21,50	0	0	In
288	k	12	2011	20	56,20	21,30	0	0	In
289	k	9	2011	29	28,40	24,56	0	0	In
289	k	5	2012	30	25,60	24,56	0	0	In
290	m	4	2012	23	16,80	20,79	0	0	In
290	m	4	2013	24	18,00	20,79	0	0	In
290	m	5	2014	25	20,10	20,79	0	0	In

291	m	9	2011	24	20,00	30,86	0	0	In
292	k	12	2011	17	18,80	23,05	0	0	In
293	m	4	2012	23	10,10	25,00	0	0	In
293	m	4	2013	24	13,00	25,00	0	0	In
293	m	5	2014	25	18,90	25,00	0	0	In
294	k	5	2010	25	18,03	23,08	0	0	In
295	m	12	2011	18	19,00	26,08	0	0	In
296	k	4	2014	19	10,90	21,30	0	0	In
297	k	5	2010	25	27,33	21,50	0	0	In
298	m	4	2012	23	16,40	22,84	0	0	In
298	m	4	2013	24	15,00	22,84	0	0	In
299	m	10	2009	25	31,35	27,16	0	0	In
300	k	12	2011	19	23,20	23,53	0	0	In
301	m	4	2012	22	16,90	22,62	0	0	In
301	m	4	2013	23	17,00	22,62	0	0	In
302	m	4	2012	24	26,40	24,94	0	0	In
302	m	4	2013	25	21,00	24,94	0	0	In
302	m	5	2014	26	26,00	24,94	0	0	In
303	m	10	2009	26	25,82	28,67	0	0	In
303	m	1	2010	27	29,17	28,67	0	0	In
303	m	6	2011	28	29,70	28,67	0	0	In
303	m	1	2012	29	25,80	28,67	0	0	In
304	k	12	2011	18	19,30	23,44	0	0	In
305	m	4	2012	24	34,50	24,27	0	0	In
305	m	4	2013	25	17,00	24,27	0	0	In
305	m	5	2014	26	13,10	24,27	0	0	In
306	k	4	2014	22	14,90	21,56	0	0	In
307	m	1	2012	27	31,10	28,22	0	0	In
308	k	5	2010	25	30,03	22,95	0	0	In
308	k	9	2011	26	27,90	22,95	0	0	In
308	k	12	2011	26	33,00	22,95	0	0	In
308	k	5	2012	27	39,60	22,95	0	0	In
309	m	10	2009	26	17,09	24,75	0	0	In
309	m	1	2010	27	4,00	24,75	0	0	In
309	m	1	2012	29	14,20	24,75	0	0	In
309	m	7	2014	31	34,30	24,75	1	0	In
309	m	2	2015	32	20,80	24,75	0	0	In
310	m	10	2009	32	21,04	24,48	0	0	In
310	m	1	2010	33	19,22	24,48	0	0	In
311	k	9	2011	22	25,60	24,92	0	0	In
312	k	5	2010	21	21,36	26,99	0	0	In

312	k	7	2010	22	24,34	26,99	0	0	In
312	k	5	2011	23	25,70	26,99	0	0	In
312	k	9	2011	22	23,60	26,99	0	0	In
312	k	12	2011	22	26,20	26,99	0	0	In
312	k	5	2012	23	30,00	26,99	0	0	In
312	k	12	2012	23	26,50	26,99	0	0	In
313	k	5	2009	21	30,78	23,23	0	0	In
313	k	10	2009	21	34,58	23,23	0	0	In
313	k	5	2010	22	26,34	23,23	0	0	In
313	k	5	2011	23	20,10	23,23	0	0	In
314	m	4	2012	25	28,20	23,16	0	0	In
314	m	4	2013	26	19,00	23,16	0	0	In
315	k	5	2010	23	27,42	20,94	0	0	In
315	k	4	2012	25	47,20	20,94	0	0	In
316	m	10	2009	23	12,64	29,08	0	0	In
316	m	1	2010	24	4,00	29,08	0	0	In
316	m	6	2011	25	19,30	29,08	0	0	In
317	k	5	2009	18	25,84	26,23	0	0	In
317	k	10	2009	18	18,21	26,62	0	0	In
317	k	9	2011	20	30,10	25,46	0	0	In
318	k	4	2012	22	35,80	21,45	0	0	In
319	k	12	2011	13	14,00	24,09	0	0	In
320	k	5	2011	19	30,90	24,09	0	0	In
321	k	12	2011	19	9,00	41,47	0	0	In
322	m	4	2012	21	10,90	19,84	0	0	In
322	m	5	2014	23	14,60	19,84	0	0	In
323	k	5	2010	31	26,00	22,86	0	0	In
323	k	9	2011	32	28,80	22,86	0	0	In
324	k	12	2011	14	13,20	20,28	0	0	In
325	m	10	2009	29	32,48	25,73	0	0	In
325	m	1	2010	30	23,19	25,73	0	0	In
326	k	10	2009	22	24,12	30,08	0	0	In
326	k	5	2010	23	27,00	30,08	0	0	In
326	k	7	2010	23	27,09	30,08	0	0	In
326	k	5	2011	24	33,50	30,08	0	0	In
326	k	9	2011	24	33,80	30,08	0	0	In
326	k	12	2011	24	44,40	30,08	0	0	In
326	k	5	2012	25	43,10	30,08	0	0	In
326	k	12	2012	25	28,40	30,08	0	0	In
327	m	4	2012	27	24,10	23,36	0	0	In
327	m	4	2013	28	26,00	23,36	0	0	In

327	m	5	2014	29	17,80	23,36	0	0	In
328	m	7	2014	28	32,70	28,79	1	0	In
328	m	2	2015	29	16,00	28,79	0	0	In
329	m	12	2011	17	13,90	19,72	0	0	In
330	m	4	2012	25	11,20	24,74	0	0	In
330	m	4	2013	26	15,00	24,74	0	0	In
330	m	5	2014	27	22,36	24,74	0	0	In
331	k	5	2010	22	24,79	21,22	0	0	In
331	k	4	2012	24	48,00	21,22	0	0	In
332	m	6	2011	22	28,60	26,32	0	0	In
333	m	12	2011	17	27,20	21,19	0	0	In
334	m	12	2011	17	29,50	26,15	0	0	In
335	k	5	2009	17	22,48	27,43	0	0	In
335	k	10	2009	17	23,82	27,43	0	0	In
336	m	10	2009	27	22,04	24,92	0	0	In
337	k	4	2012	27	53,90	20,56	0	0	In
338	k	5	2010	19	21,24	18,51	0	0	In
339	k	4	2014	25	16,70	21,33	0	0	In
340	k	5	2009	18	24,03	25,43	0	0	In
340	k	10	2009	18	25,37	25,43	0	0	In
340	k	5	2011	20	23,40	25,43	0	0	In
341	m	12	2011	18	27,90	26,64	0	0	In
342	m	7	2014	26	37,00	24,62	1	0	In
342	m	2	2015	27	14,00	24,62	0	0	In
343	k	5	2011	15	29,20	23,44	0	0	In
343	k	9	2011	15	24,60	23,44	2	0	In
343	k	12	2011	15	22,50	23,44	0	0	In
343	k	5	2012	16	28,10	23,44	0	0	In
343	k	12	2012	16	22,90	23,44	0	0	In
344	m	10	2009	25	44,07	26,04	0	0	In
344	m	1	2010	26	48,00	26,04	0	0	In
344	m	1	2012	28	46,60	26,04	0	0	In
344	m	7	2014	30	36,50	26,04	1	0	In
344	m	2	2015	31	7,45	26,04	0	0	In
345	m	4	2012	29	15,90	22,20	0	0	In
345	m	4	2013	30	18,00	22,20	0	0	In
345	m	5	2014	31	13,90	22,20	0	0	In
346	k	12	2011	16	20,70	21,08	0	0	In
347	k	5	2010	26	30,03	20,52	0	0	In
348	m	7	2014	29	36,40	26,30	1	0	In
348	m	2	2015	30	11,40	26,30	0	0	In

349	k	5	2010	25	28,93	21,50	0	0	In
349	k	4	2012	24	35,90	21,50	0	0	In
350	m	10	2009	34	28,91	29,33	0	0	In
350	m	1	2010	35	30,71	29,33	0	0	In
351	m	12	2011	17	17,70	28,40	0	0	In
352	k	4	2012	24	21,10	20,60	0	0	In
353	k	4	2012	29	19,60	21,20	0	0	In
353	k	4	2014	31	15,80	21,20	0	0	In
354	k	4	2012	29	15,80	20,44	0	0	In
355	m	12	2011	17	10,90	28,01	0	0	In
356	k	5	2009	24	41,69	22,95	0	0	In
356	k	10	2009	24	25,94	22,95	0	0	In
357	k	12	2011	22	33,50	21,30	0	0	In
358	m	12	2011	17	35,90	26,37	0	0	In
359	k	5	2010	23	27,47	20,20	0	0	In
360	m	1	2012	21	51,10	26,81	0	0	In
361	k	12	2011	16	9,10	20,00	0	0	In
362	m	10	2009	31	21,00	27,45	0	0	In
362	m	1	2010	32	15,89	27,45	0	0	In
362	m	6	2011	33	24,80	27,45	0	0	In
362	m	7	2014	36	30,60	27,45	1	0	In
362	m	2	2015	37	23,10	27,45	0	0	In
363	m	9	2011	27	21,80	29,03	0	0	In
364	k	4	2014	20	7,14	19,45	0	0	In
365	m	12	2011	16	18,20	24,80	0	0	In
366	k	12	2011	23	17,80	24,02	0	0	In
367	m	12	2011	17	18,50	28,28	0	0	In
368	m	7	2014	26	39,80	23,89	1	0	In
369	m	1	2012	32	27,90	25,25	0	0	In
369	m	7	2014	34	29,80	25,25	1	0	In
369	m	2	2015	35	12,10	25,25	0	0	In
370	m	10	2009	30	22,18	27,46	0	0	In
370	m	1	2010	31	15,84	27,46	0	0	In
370	m	6	2011	32	17,80	27,46	0	0	In
370	m	1	2012	33	15,60	27,46	0	0	In
371	k	4	2012	21	37,10	19,33	0	0	In
371	k	4	2014	23	25,90	19,33	0	0	In
372	m	6	2011	26	24,20	25,76	0	0	In
372	m	1	2012	27	24,70	25,76	0	0	In
373	k	4	2014	19	19,30	22,35	0	0	In
374	k	10	2009	20	19,68	25,91	0	0	In

374	k	5	2010	21	21,77	25,91	0	0	In
374	k	5	2011	22	39,00	25,91	0	0	In
374	k	9	2011	22	18,80	25,91	0	0	In
374	k	12	2011	22	20,00	25,91	0	0	In
374	k	5	2012	23	29,70	25,91	0	0	In
375	k	4	2012	28	54,90	21,78	0	0	In
376	m	1	2012	32	14,10	26,85	0	0	In
377	m	4	2013	25	27,00	24,48	0	0	In
378	m	4	2012	29	25,30	23,50	0	0	In
378	m	4	2013	30	33,00	23,50	0	0	In
378	m	5	2014	31	16,00	24,48	0	0	In
379	m	1	2012	32	20,90	23,55	0	0	In
380	m	4	2012	23	16,00	26,28	0	0	In
380	m	4	2013	24	13,00	26,28	0	0	In
380	m	5	2014	25	15,30	23,50	0	0	In
381	m	5	2014	31	21,50	26,80	0	0	In
382	m	5	2014	31	18,60	21,26	0	0	In
383	m	10	2009	33	18,82	25,66	0	0	In
384	m	4	2013	23	18,00	22,75	0	0	In
385	m	4	2012	29	21,20	22,69	0	0	In
385	m	4	2013	30	20,00	22,69	0	0	In
385	m	5	2014	31	18,50	22,69	0	0	In
386	k	5	2010	25	29,70	22,48	0	0	In
387	m	4	2012	24	21,30	23,46	0	0	In
387	m	4	2013	25	17,00	23,46	0	0	In
387	m	5	2014	26	16,60	23,46	0	0	In
388	k	5	2010	22	30,40	20,00	0	0	In
389	k	5	2010	20	28,87	22,89	0	0	In
389	k	4	2012	22	50,30	22,89	0	0	In
389	k	4	2014	24	37,30	22,89	0	0	In
390	k	4	2014	20	7,73	21,62	0	0	In
391	m	4	2012	24	8,90	22,61	0	0	In
391	m	4	2013	25	14,00	22,61	0	0	In
391	m	5	2014	26	12,10	22,61	0	0	In
392	k	5	2009	28	19,33	36,75	0	0	In
392	k	5	2010	29	15,24	36,75	0	0	In
393	k	12	2011	18	16,20	22,66	0	0	In
394	k	12	2011	15	48,10	22,06	0	0	In
395	m	10	2009	21	28,54	25,25	0	0	In
395	m	1	2010	22	27,18	25,25	0	0	In
395	m	6	2011	23	29,00	25,25	0	0	In

395	m	1	2012	24	21,50	25,25	0	0	In
396	k	10	2009	22	25,06	24,22	0	0	In
397	k	5	2011	19	8,90	24,20	0	0	In
397	k	9	2011	19	30,30	24,20	0	0	In
397	k	12	2011	19	33,90	24,20	0	0	In
397	k	5	2012	20	49,70	24,20	0	0	In
397	k	12	2012	20	37,50	24,20	0	0	In
398	m	5	2014	37	20,80	22,00	0	0	In
399	m	4	2013	25	15,00	24,75	0	0	In
400	m	1	2012	28	31,70	25,25	0	0	In
401	k	5	2010	23	30,60	23,99	0	0	In
402	k	4	2014	27	28,70	23,99	0	0	In
403	m	4	2012	22	28,10	21,50	0	0	In
403	m	4	2013	23	18,00	21,50	0	0	In
403	m	5	2014	24	19,10	21,50	0	0	In
404	k	4	2012	30	10,00	21,72	0	0	In
405	m	7	2014	34	38,60	26,87	1	0	In
405	m	2	2015	35	25,50	26,87	0	0	In
406	m	9	2011	21	21,60	30,10	0	0	In
407	m	4	2012	35	27,10	24,50	0	0	In
408	m	4	2012	24	18,10	26,82	0	0	In
408	m	4	2013	25	13,00	26,82	0	0	In
409	m	10	2009	25	21,28	27,61	0	0	In
409	m	1	2010	26	14,49	27,61	0	0	In
409	m	6	2011	27	24,00	27,61	0	0	In
410	m	12	2011	14	13,00	28,40	0	0	In
411	m	4	2013	25	12,00	24,45	0	0	In
412	m	4	2012	33	33,80	22,28	0	0	In
